# Is There Evidence for Myelin Modeling by Astrocytes in the Normal Adult Brain?

**DOI:** 10.3389/fnana.2017.00075

**Published:** 2017-09-04

**Authors:** Alfredo Varela-Echevarría, Víctor Vargas-Barroso, Carlos Lozano-Flores, Jorge Larriva-Sahd

**Affiliations:** Department of Developmental Biology and Neurophysiology, Instituto de Neurobiología Universidad Nacional Autónoma de México Querétaro, Mexico

**Keywords:** astrocytic process, neuropil, myelin remodeling, lysosome, secretion

## Abstract

A set of astrocytic process associated with altered myelinated axons is described in the forebrain of normal adult rodents with confocal, electron microscopy, and 3D reconstructions. Each process consists of a protuberance that contains secretory organelles including numerous lysosomes which polarize and open next to disrupted myelinated axons. Because of the distinctive asymmetric organelle distribution and ubiquity throughout the forebrain neuropil, this enlargement is named paraxial process (PAP). The myelin envelope contiguous to the PAP displays focal disruption or disintegration. In routine electron microscopy clusters of large, confluent, lysosomes proved to be an effective landmark for PAP identification. In 3D assemblies lysosomes organize a series of interconnected saccules that open up to the plasmalemma next to the disrupted myelin envelope(s). Activity for acid hydrolases was visualized in lysosomes, and extracellularly at the PAP-myelin interface and/or between the glial and neuronal outer aspects. Organelles in astrocytic processes involved in digesting pyknotic cells and debris resemble those encountered in PAPs supporting a likewise lytic function of the later. Conversely, processes entangling tripartite synapses and glomeruli were devoid of lysosomes. Both oligodendrocytic and microglial processes were not associated with altered myelin envelopes. The possible roles of the PAP in myelin remodeling in the context of the oligodendrocyte-astrocyte interactions and in the astrocyte's secretory pathways are discussed.

## Introduction

Understanding cellular domains has been an essential preliminary step to define functional properties inherent to each cell type. An increasing amount of evidences for the existence of cytoplasmic domains in astrocytes (ACs) has recently been accumulated. Earlier descriptions of the AC established two broad subtypes as identified in silver-impregnated specimens. According to the cell location, size, and structure of its radial processes, two AC subtypes are identified: protoplasmic and fibrous ACs. Protoplasmic ACs are commonly associated with nuclei and neuron layers, whereas fibrous ACs lie between axonal fascicles in the white matter. While the overall branching and distribution of AC processes is radial, thereby occupying rounded to oval fields (Del Río Hortega, 1914; Butt and Ransom, 1993), regional variations of astrocytic processes (APs) that reside in elliptical and/or overlapping fields are frequent (Butt et al., 1994; López-Hidalgo et al., 2016). Although primary APs generally follow a straight course, they each originate numerous shorter formations whose submissive accommodation among the neighboring neural and stromal elements imprint to them assorted, frequently, unpredictable shapes (Göbel, 1966; Valverde and López-Mascaraque, 1991). Perhaps the best-known exception regarding the signed pleomorphism of the APs is where they interact with the outer aspect of the blood vessels. In fact, one or a set of APs terminate in distinct cytoplasmic expansions, or end-feet, that surround capillary blood vessels, thereby creating a continuous sheet of APs lining the blood vessels throughout most areas of the central nervous system. Sites of the brain where capillaries are devoid of this astrocytic cuff are commonly permeable to dyes and other high molecular substances that are otherwise kept in the vascular compartment. This association of the end-feet with the capillary wall provides the structural basis for the blood-brain barrier (Brightman and Reese, 1969; Peters et al., 1976; Broadwell and Sofroniew, 1993). Additional support to the functional role of AC's end-feet is their reciprocal inductive influences with the under laying vascular endothelium (Sperri et al., 1997). Furthermore, localization of aquaporin in the AC end-feet has been implicated in the pathogenesis of neuromyelitis optica autoimmune (Jarius and Wildeman, 2010), a histopathological variant of multiple sclerosis. Another instance recognized earlier by Ramón y Cajal (1904) relates to a group of APs termed pericellular pedicles (“pedículos pericelulares”) that ramify to surround neighboring neuronal perikarya (Del Río Hortega, 1914). More recent studies have shown that this pericellular framework influences the plasticity of synaptic inputs that arrive to the neuron's soma (Garcia-Segura et al., 1994). It has been further demonstrated that the sites of interaction between the AC processes and synaptic terminals noted earlier (Göbel, 1966), define substrates for chemical and functional reciprocal neuron-glia interactions, the so-called tripartite synapses (Araque et al., 1999; Ventura and Harris, 1999; Witcher et al., 2010). Furthermore, glycogen that is stored and provided by the AC exhibits unique regional and cytoplasmic patterns of distribution (Cali et al., 2016) that concentrate next to axon boutons and postsynaptic spines, in order of frequency, supporting the notion that ACs modulate chemical synaptic transmission. Lastly, there is a set of cytoplasmic digitations arising from the AC perikaryon (see Del Río Hortega, 1914) or the end-foot proper, termed perinodal processes that directly interact with the Ranvier node to share gap junctions with the bounding paranodal loops of the oligodendrocyte (Ol; Black et al., 1984; Waxman and Black, 1984; Black and Waxman, 1988). The possibility that this AC-Ol interaction plays a role in myelin homeostasis has been put forth (Hildebrand et al., 1993).

Astrocytes play a key role in a variety of cellular responses including: cell death (He et al., 2006), developmental and adult synaptic pruning (see Chung et al., 2014), nerve degeneration (Fried and Hildebrand, 1982; Hildebrand, 1989), experimental lesioning of neurons and axon terminals (Price and Powell, 1970a; Larriva-Sahd, 2008; Marcellino et al., 2012; Anderson et al., 2016), and phagocytosis of debris (see Tasdemir-Yilmaz and Freeman, 2014; Iram et al., 2016). The AC is also a cardinal cell type for the ontogenetical process of myelin synthesis by the Ol. Thus, the AC interacting directly with the Ol precursor cell, promotes viability (Corley et al., 2001) of this cell type to further migrate (Bauman and Pham-Dinh, 2001) and differentiate to the myelin-synthesizing Ol (see Clemente et al., 2013). It is also accepted that the AC proper is directly involved in myelin retrieval in the plasticity of the developing nervous system, leading to the possibility that ACs are responsible for the process of myelin remodeling during the adulthood (Hildebrand and Aldskogius, 1976). Indeed, available studies in normal adult mammals showed that microglia and perivascular, and astroglial cells, in decreasing frequency, interact and internalize fragmented myelin envelopes (Hildebrand and Aldskogius, 1976). Furthermore, interaction of the normal AC bearing lysosomes positive to acid hydrolases with altered myelinated fibers, was noted earlier (Hildebrand, 1971; Hildebrand and Skoglund, 1971). To our knowledge these authors demonstrated by first time that lysosome-bearing AC coexists with altered myelinated fibers in the normal brain. Thus far, it is uncertain how lysosomes interact with either phagocytized or extracellular myelin. An important recent study of the amphibian optic nerve defined that ACs remodel the redundant myelin envelope of axons that results from metamorphosis (Mills et al., 2015). While in the normal mammalian central nervous system the Ol is readily involved in myelin sorting at the molecular domain (Maier et al., 2008) the role of Ols, microglia, and macrophages in myelin clearance appears to be reserved to pathological, pharmacological or experimental (Nathaniel and Pease, 1963; Hildebrand et al., 1993, 1994) conditions leading to myelin damage. Present study combines cyto-, immuno-histochemical, and electron microscopic techniques to define how astrocytic processes interact with myelin. Then, series of ultrathin sections were used to depict a 3D structure at the subcellular level. Regular identification of a set of APs based on its vesicular contents and its association with myelinated fibers piercing the neuropil confirmed their ubiquitous distribution. Present observations define the existence of a ubiquitous group of APs containing numerous lysosomes and other secretory organelles (see Zorec et al., 2017) that interact with the altered myelin envelope of nearby axons, suggesting that they are involved in normal myelin remodeling in the adult forebrain. Experimental work describing the APs response to axon degeneration will be separately presented.

## Materials and methods

### Animals

Normal adult albino rats and mice where utilized. Mice belonged the colony *FVB/N-Tg (GFAP-EGFP)GFEA-FKi* that display EGFP (eGFP) fluorescence in astrocytes from multiple areas of the CNS (Nolte et al., 2001). Founders of the colony [GFAP-EGFP] were kindly provided by Dr. Helmut Kettenmann (Max Delbruck Center for Molecular Medicine, Berlin, Germany). All animals were males of 10 weeks of age that were raised in a pathogen-free colonies in accordance with animal care policies in our vivarium. Both the study and endeavor of animal handling and experimental manipulations were reviewed and approved (Code number: 2016-60) by Comité de Ética en Investigación, Instituto de Neurobiología, Universidad Nacional Autónoma de México. This committee was appointed by our director and consisted of five professional scientific researchers trained in ethics and familiar with the current international guide-lines of Biomedical research (see Ethics Committee SfN home-page). Animals were killed by vascular aldehyde perfusion (vide infra) under deep anesthesia (i.e., 30 mg/kg pentobarbital) or decapitation as separately described below.

### Histofluorescence and immunohistochemestry

To label acidic organelles (i.e., lysosomes) LysoTracker red (Invitrogen-Molecular Probes, Carlsbad, CA, USA) was used as described elsewhere (Weis et al., 2014). Briefly, seven *GFAP-EGFP* (eGFP) mice were decapitated and brains were immediately removed from the skull. Then, under the dissecting microscope, each brain was transversally divided into three thirds with a razor blade. Blocks of tissue were incubated for 5 min in a solution of phosphate buffered saline (0.1M, pH 7.4) and LysoTracker (1:400). Following a brief rinse in PBS, blocks of tissue were left in a 4% paraformaldehyde fixative diluted in the same vehicle for 12 h at 4°C. Tissues were transferred to 30% sucrose solution for 2 days and subsequently embedded in Tissue-Tek (OCT, Sakura) to obtain 50 μm sagittal sections with a cryostat (Leica Biosystems). Sections were transferred to PBS, blocked in PBS containing 5% normal goat serum (NGS) and 0.1% Triton X-100 for 45 min and incubated overnight at 4°C in myelin basic protein (MBP) antibody (Molecular Probes, Invitrogen) diluted 1:1,000 in PBS. Then, sections were immersed in a secondary rabbit Cy5 diluted 1:1,000 in PBS for 2 h. Following a 5-min wash in PBS, sections were incubated in DAPI diluted 1:4,000 for 5 min and mounted in Mowiol medium. Observations and image acquisition were performed using a Zeiss 780 LSM confocal microscope. Confocal micrographs were acquired at 0.37 μm interval at 1,024 × 1,024 pixel resolution and further processed and edited with the “Image J” and “Adobe Photoshop” software, respectively. Illustrative 3D videos from **Figures 2B,D**, were obtained with the “Amira” software (Supplemental Material).

### Histochemistry and cytochemistry

As virtually all lysosomal enzymes are acidic hydrolases (DeDuve, 1983), specimens from five brains were incubated with beta-glycerolphosphate, a well-known substrate to these enzymes (Welsh, 1966; Nichols et al., 1971; Hildebrand, 1982). An alternative technique was utilized, as the enzyme products of the incubation are both chromogenic and electron opaque (Hildebrand and Skoglund, 1971; Bencosme et al., 1979). Thus, sites that are positive to the reaction are visualized under both a conventional light- and an electron microscopes. To accomplish this, five adult rat brains were used. Animals were deeply anesthetized and perfused through the left ventricle with 250 ml of Karnovsky's fixative cooled at 4°C. The fixative consisted of a 4% paraformaldehyde and 2% glutaraldehyde dissolved in 0.1M sodium cacodylate buffer at pH 7.4. Then, each brain was removed from the skull and transferred to fresh Kanrnovsky fixative, where it remained overnight at 4°C. The next day each brain was cut with a vibratome in the sagittal plane at 100 μm of nominal thickness. Sections were incubated in a medium at low-pH containing a beta-glycerolphosphate substratum as detailed elsewhere (Bencosme et al., 1979). Sections incubated in absence of the substratum were left as controls. Demonstrative sections were chosen for light microscopy on the basis of their completeness; then, they were mounted in glass slides and coverslipped with glycerin or further processed for electron microscopy.

### Electron microscopy

Brains from five adult rats were used for this part of the study. Rats were deeply anesthetized and perfused through the left ventricle with Karnovsky's fixative and processed as previously described (Larriva-Sahd, 2006, 2008). Small tissue blocks (~1 mm per side) from the olfactory bulb, ventral hippocampus, and frontal and parietal isocortices were dissected-out from these brains and from sections that had previously been incubated for cytochemistry to acid phosphatases (vide supra). Samples of tissue were postfixed for 1 h in 1% osmium tetraoxide dissolved in 0.1 M cacodylateHCl buffer; then they were dehydrated in graded acetone, and embedded in epoxy resin. Additional tissue blocks from the ventro-medial hypothalamic and medial preoptic nuclei, and the piriform cortex were taken from prior work and processed as described underneath. One micrometer-thin sections were obtained from the tissue blocks in a Leica ultramicrotome utilizing with glass knives. The sections were stained with toluidine blue and coverslipped. Areas to be studied from these blocks included the part of the gray matter with higher occurrence of myelinated fibers and lysosome-bearing processes (see Hildebrand and Skoglund, 1971) as it follows: Layers VI and V of the cerebral cortex (Tomassy et al., 2014), Layer I of the piriform cortex (Vargas-Barroso and Larriva-Sahd, 2013), outer aspects of the medial preoptic (Larriva-Sahd and Gorski, 1987) and ventromedial hypothalamic (Larriva-Sahd et al., 1995) nuclei, intersection of the olfactory bulb cortex and medulla (Larriva-Sahd, 2014), and the ilium of the dentate gyrus. Silver sections at 70 nm of nominal thickness were obtained with a Leica ultramicrotome and mounted on copper grids. Thinner sections approximating 50–55 nm of thickness were utilized to depict organelle interactions at magnifications above of 50,000 x. In either case, sections were sequentially stained with uranium and lead salts and they were observed under a JOEL 1010 electron microscope operated at 80 kV equipped with an automated goniometer.

### Ultrastructural reconstructions and morphometry

Thirty-one series of thin sections encompassing 2,141 sections (Table 1) were utilized to perform 3D reconstructions as described in prior work (Larriva-Sahd, 2014). To test the effect of lysosomal structure in astrocytic processes associated with myelinated axons, 10 additional series, including four pyknotic cells in the rostral migratory stream (Schmechel, 1999; He et al., 2006; Iram et al., 2016), four tripartite synapses (Araque et al., 1999), and three glomeruli (Price and Powell, 1970b) were included. Areas of interest were sequentially photographed from the electron microscope with a Gatan digital camera. Images from each series were aligned and assembled with a free Reconstruct software (http://synapses.clm.utexas.edu/tools/reconstruct/reconstruct.stm.). Astroglial processes containing rough endoplasmic reticulum, mitochondria, Golgi apparatus, electron-dense granules, and intermediate filaments, termed here “paraxial processes” (PAPs), were identified and then successively photographed throughout the series (Table 1). Axonal and lysosomal dimensions were obtained from the digital micrographs that were utilized for the 3D reconstructions aided by a PC running the Reconstruct software. The transverse diameter of all myelinated fibers interacting with the astrocytic process was defined by direct measurements taken from the Reconstruct software and expressed as the mean axonal diameter per brain area to be studied. A minimum of 30 axons and 800 electron-dense granules was considered for each case.

**Table 1:** Specimen sources, sampling, and axon and organelle frequency in serial sections.

		**Number of series**	**Number of sections**	**Myelinlysosome apposition**	**Mean axonmyelin diameter (μm)**	**Lysosome occurrence**	Paraxial processes^**^
Olfactory bulb	Gr.C.L.	7	542	5	0.68	++++	17.5
	S.L.	9	680	8	0.95	++++	23
Frontal isocortex (Layer VI)		3	153	2	1.2	++	11.5
Parietal isocortex (Layer VI)		3	204	2	1.34	++	9.8
Ammon's Horn (ilium of the dentate nucleus)		4	240	3	1.02	++	13
Medial preoptic nucleus		2	90	1	0.78	++	11
Ventromedial hypothalamic nucleus		3	152	2	0.90	+++	11
Total		31	2,141		0.98 μm^*^		

*Each + means 5 secondary lysosomes per reconstruction*.

* *, Mean transverse axis*.

** *, Percentage of the total of reconstructed PAPs per brain area*.

### Volume density of axons, paraxial, and oligodendroglial processes

The incidence of glial processes and myelinated axons in series of 40 serial sections (see in Section Electron Microscopy) was quantified into three forebrain areas. Specimens from olfactory bulb, temporal isocortex, and ventromedial hypothalamic nucleus were submitted to a modified protocol as described elsewhere (Granados-Rojas et al., 2002). Briefly, numbers of PAPs (see Section A Set of Astrocytic Processes Contain Secretory Organelles) and oligodendrocytic processes (see Section Oligodendrocytes and Microglia) were counted through the series at the electron microscope operated at a variable magnification ranging from 5,000 to 40,000x. Double counts were prevented by omission of structures overlapping the X-Y site in the successive sections. Then, the volume of studied tissue was calculated by multiplying the total area of each section by its nominal thickness (i.e., 70 nm) and, then, by the number of sections. For comparison purposes a total volume of 70,000 μm^3^ was considered. As three series per brain area were included a total volume of 21 × 10^4^ μ^3^m was studied. Last, the number of myelinated fibers per volume was calculated as it follows. Six electron micrographs at 3,000x magnification were obtained from random areas of the first and last sections. From each digital micrograph the number of axons was obtained with the “Axonseg” open software (Zaimi et al., 2016). The photographed area was normalized to the total area per section and expressed as the mean number of fibers of the first and the last section in the volume of the series.

### Terminology

Terms used throughout the text to designate frequency of occurrence of the structures in more than two-thousand sections (Table 1) under the electron microscope have the following connotations: “Frequent,” “usual,” or “not infrequently” = present in all or in most sections; “relatively common” = present in at least three or more sections per brain area; “Occasional(ly)” or “uncommon(ly)” = less than three per brain area; “rare” = one to three times throughout the study in a given brain area.

The well-known limitation to define a granule containing electron-dense, often pleomorphic, material as a lysosome (Fawcet, 1981; DeDuve, 1983) is prevented here with a straightforward nomenclature. Thus, whenever a suspected lysosome is observed it is termed “electron-dense granule” or “electron-opaque granule.” Meanwhile, the term “lysosome” is applied to a granule that has an identical structure to the one visualized first in specimens incubated with the substratum (i.e., beta-glycerolphosphate) to detect acid phosphatases. In line with an earlier notion (Holtzman and Dominitz, 1968; Fawcet, 1981) the presence of membranous and/or lipid droplet inclusions in presumptive lysosomes is used as a structural landmark to designate “secondary lysosomes” throughout the text. The term “lysosome” is also used to designate granules positive to the Lyso-Tracker reagent under the confocal microscope (Weis et al., 2014). Finally, the terms “apoptotic cell” and “apoptosis” apply exclusively to shrunken cells in the olfactory bulb medulla which have an overall electron-opaque appearance and varying degrees of nuclear and/or cytoplasmic disruption. These characteristics match with previous observations of apoptotic cells during adult neurogenesis (He et al., 2006), and they are thoroughly analyzed elsewhere (Schmechel, 1999).

## Results

Our observations are directed toward the neuropil of various forebrain areas, with an emphasis on the astrocytic processes contained therein. Since cytological characteristics of the AC and neuropil proper have been extensively presented elsewhere (Göbel, 1966; Butt and Ransom, 1993; Butt et al., 1994; Ventura and Harris, 1999; Witcher et al., 2010), only a brief account will be provided for structural context.

**Figure 1:**
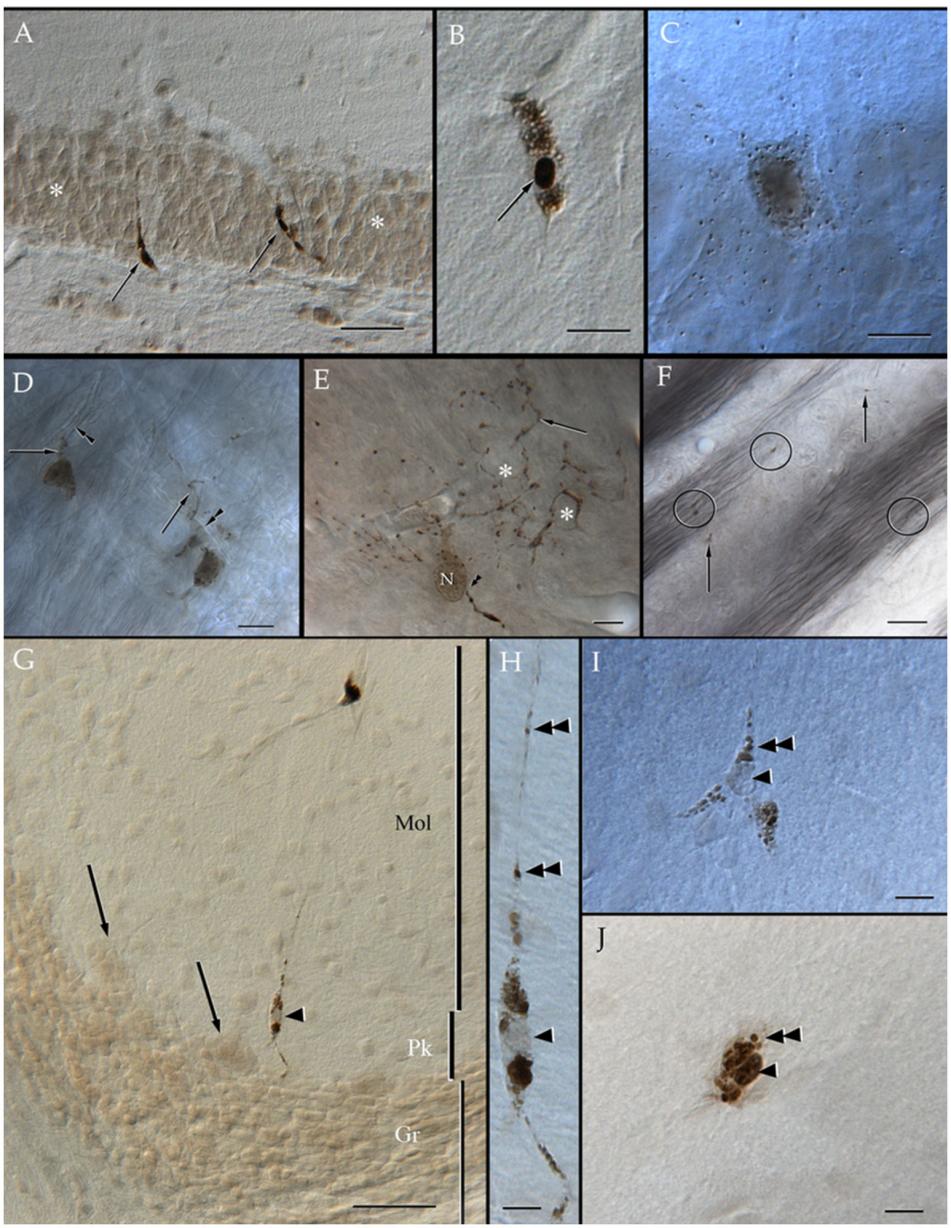
Distribution of acid phosphatase positive cells and lysosomes in the neuropil and white matter. **(A)** Ilium of the dentate gyrus. Presumptive microglial cells (arrows) containing numerous lysosomes. Note that cell processes course between granule cells (asterisks). **(B)** Microglia with abundant lysosomes at either pole of the nucleus (arrow) that is also positive to the histochemical reaction. **(C)** A presumptive pyramidal cell in layer VI of the frontal cortex. Note that numerous lysosomes are scattered in the perikaryon. **(D)** Two presumptive microglial cells in the neuropil of the deep bulbar white matter of the olfactory bulb. To highlight is the linear arrangement of lysosomes in the cell's processes (arrows) that appose unstained, myelinated axons (arrowheads). **(E)** Cell in the glomerular layer of the olfactory bulb displaying an overall diffuse positivity to the reaction products. Note the varicose appearance of processes due to rows of lysosomes. As processes bend repeatedly they bound squared- or polygonal-shaped (asterisks) areas of the neuropil. Arrowheads, root of a proximal process; N, cell nucleus. **(F)** Granule cell layer of the olfactory bulb. Isolated lysosomes can be seen among the bundles of myelinated fibers (circles) and between the adjacent clusters of granule cells (arrows). **(G)** Radial glial cell whose soma (arrowhead) lies in the Purkinje cell layer of the cerebellum; in the upper part, a microglial cell can also be seen. Mol, molecular layer; Pk, Purkinje cell layer; Gr, granule cell layer of the cerebellar cortex. **(H)** High magnification micrograph from the Bergman radial glial cell shown in “**G**.” To note is the pale cell nucleus (arrowhead) and the rows (arrowheads) of lysosomes along the ascending process. **(I)** A presumptive astrocyte whose pale nucleus (arrowhead) is surrounded by numerous lysosomes that penetrate proximal processes (double arrowheads). **(J)** A probable microglia having a dark nucleus, as well as tightly packaged lysosomes (double arrowhead) positive to the reaction. Adult rat brain. Normarski optics Scale bars = 100 μm in **(A,G)**, 10 in **(B–D)** and **(H–J)**.

**Figure 2:**
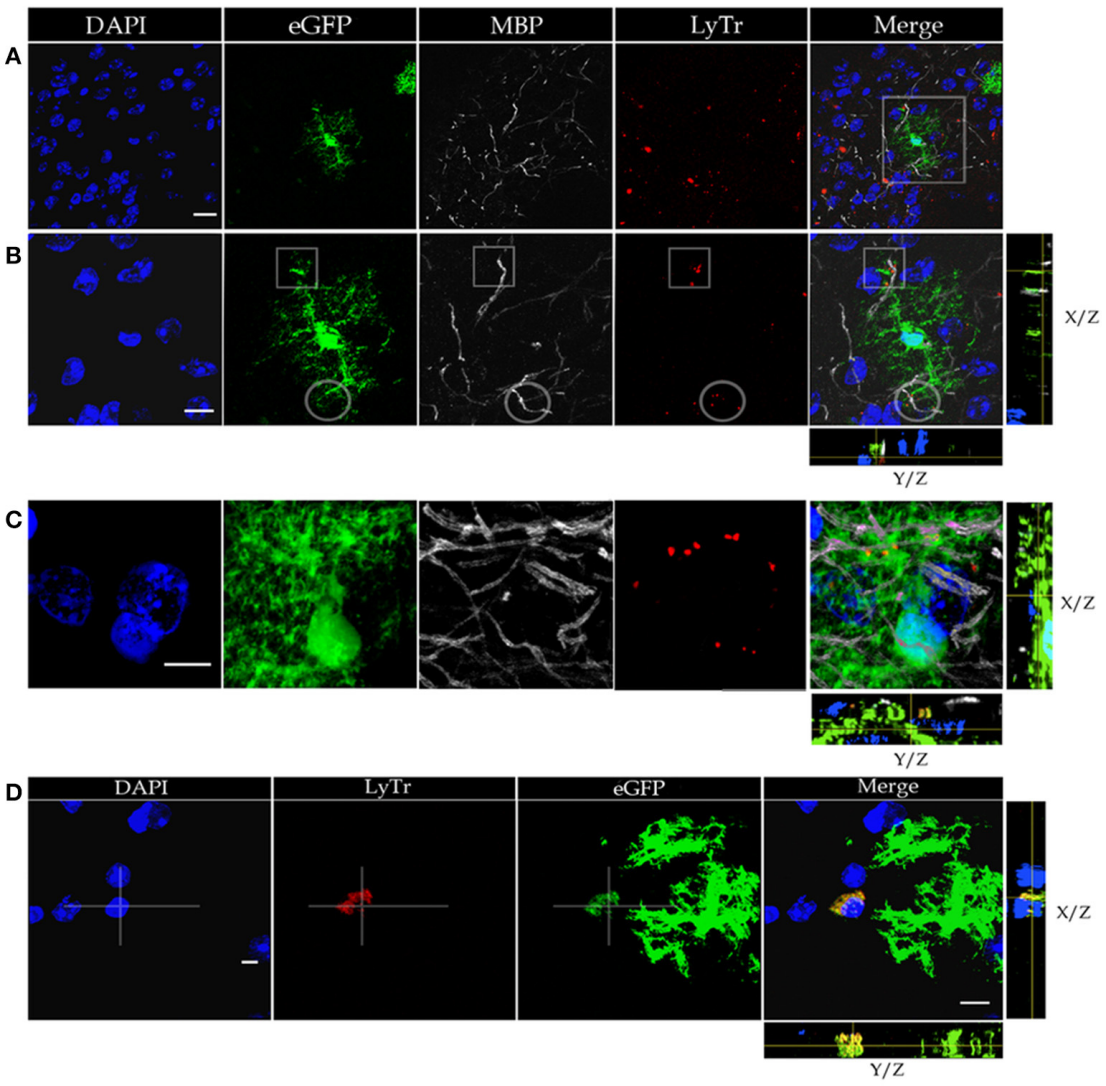
Confocal microscopy of specimens from a mouse expressing green fluorescence protein (eGFP) in astrocytes, immuno-stained to myelin basic protein (MBP), lysosomes positive to Lyso-Tracker (LyTr-positive), and counterstained with DAPI in the olfactory bulb medulla **(A,B,D)** and layer VI of the frontal isocortex **(C)**. **(A)** Low magnification view. Note that some lysosomes LyTr-positive throughout the neuropil over impose sites of eGFP and MBP (Merged) fluorescence. **(B)** Higher magnification view to the area framed in “**A**.” 3D video is avialable as Video 1 in “Supplemental Material.” Co-labeling to eGFP, MBP, and LyTr the astrocyte distal processes. **(C)** Note the discreet sites of co-labeling of the astrocyte processes, myelinated axons, and lysosomes. **(D)** Cluster of LyTr-positive lysosomes (red) co-labeled with eGFP fluorescence surrounding a shrunken nucleus (DAPI) of a presumptive dead cell. Note the numerous astrocytic processes eGFP-positive and LyTR-positive lysosomes. 3D video is avialable as Video 2 in “Supplemental Material.” Scale bars = 10 μm.

Specimens submitted to enzyme histochemistry (Figure 1), or Lyso-Tracker fluorescence (Figure 2), depict presumptive cell processes containing numerous rounded lysosomes that have assorted sizes from 0.3 to 0.5 μ in diameter. Lysosomes are distributed throughout the neuropil and white matter with a seemingly higher incidence in the former and at sites of transition between them (Figure 1C), as noted earlier in specimens from other mammals (Hildebrand and Skoglund, 1971; Hildebrand, 1982). Processes in the gray matter contain rows of lysosomes paralleling myelinated fibers that pierce through it, although clusters of three or more granules are not infrequent (Figures 1C,E). In both cases lysosome-laden processes bind polygonal or square-shaped areas of the neuropil (Figure 1E). Lysosomes are sparse within the white matter and lie between unstained bundles of myelinated fibers (Figure 1F). Occasional cells exhibit both nuclear and cytoplasmic labeling to acid phosphatase. Specifically, the nuclei of presumptive ACs (Figure 1I) and Bergman's radial glia (Figures 1G,H) are pale and homogeneous, contrasting the dark granular appearance of the cytoplasm due to numerous clusters of lysosomes. In appropriate sections, rows of lysosomes may be seen in the AP. Microglia exhibits a central dark nucleus with numerous lysosomes filling the oval cytoplasm (Figures 1B,J). Neurons usually contain an either weak or absent nuclear reactivity; however, a moderate to large number of small (i.e., <0.2 μm) lysosomes are identified throughout the perikaryon and proximal dendrites (Figure 1C).

### Astrocytic processes appose to myelinated fibers

To define the distribution of lysosomes in the AC cytoplasm, we utilized specimens from a mouse strain displaying eGFP positivity in this cell type, and we further processed them to visualize both lysosomes and myelinated fibers (Figure 2). This is accomplished through Lyso-Tracker fluorescence followed by incubation with the myelin basic protein (MBP) antibody (Figures 2A–C). It is noted that the eGFP-positive, distal astrocytic processes in such specimens regularly co-fluoresce with scattered or clustered lysosomes. These double labeled elements co-label focally with MBP-positive fibers, thereby suggesting an actual apposition of distal APs with the latter, although a similar pattern of co-labeling is occasionally observed next to the perikaryon. In sections throughout the olfactory bulb medulla, lysosomes contained by presumptive APs accumulate about pyknotic (Figure 2D) or fragmented nuclei. Since the photic light and confocal microscopes provide a limited resolution of cell membranes and organelles, or interactions between them are frequently overlapped, appropriate specimens were used for the electron microscope as described next.

### A set of astrocytic processes contain secretory organelles

**Figure 3:**
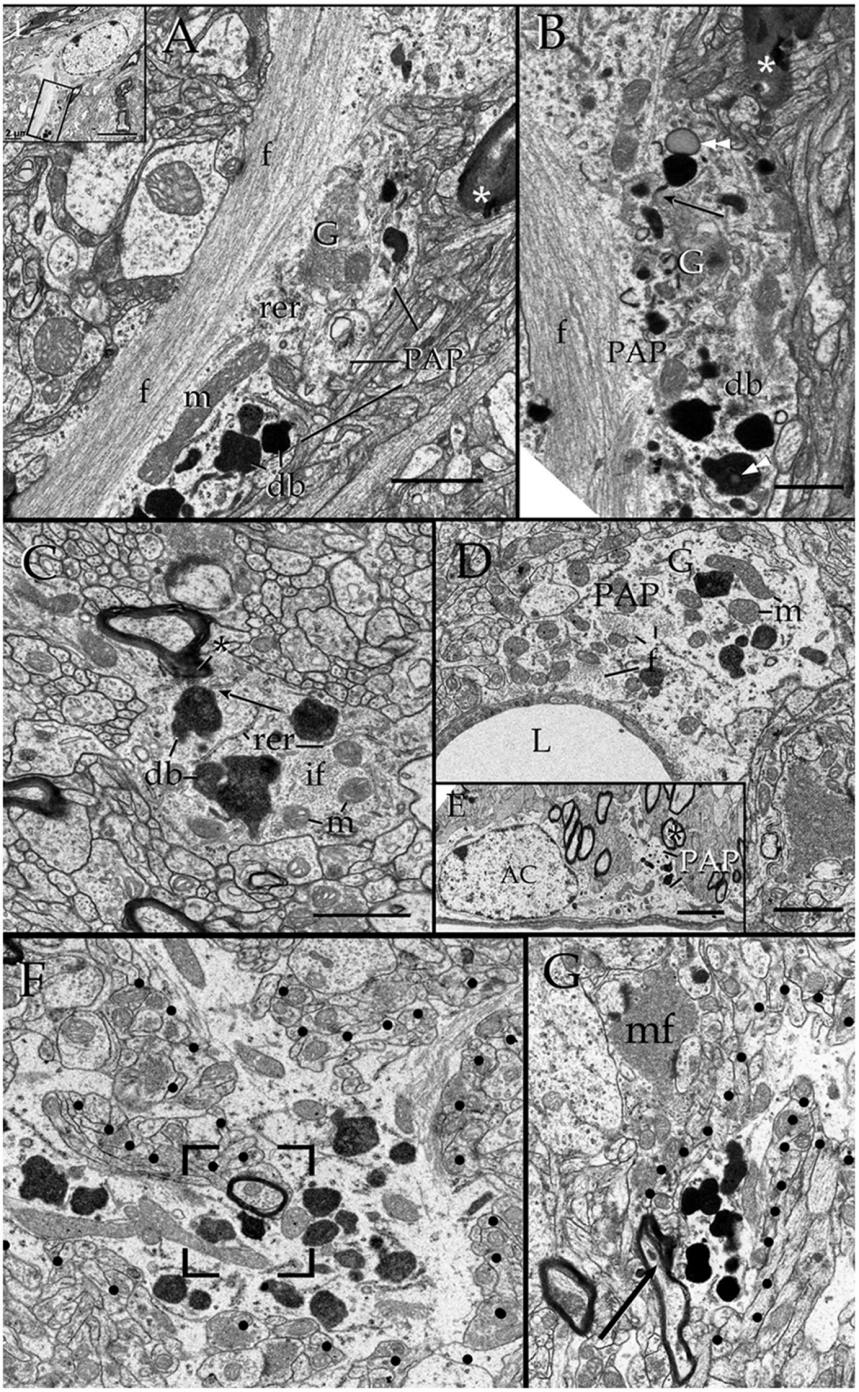
Electron micrographs from astrocytic paraxial processes in various areas of the adult rat forebrain. **(A)** Olfactory bulb medulla. Proximal process from that part of the astrocyte boxed in “i” (low magnification of the astrocyte whose process is shown in “**A**”). A thick, descending bundle of intermediate filaments (f) penetrates a paraxial process (PAP) that contains a large mitochondrion (m), Golgi apparatus (G), and several large electron-dense granules (db). Note the myelinated fiber (asterisk) next to the process. **(B)** A higher magnification view of a successive section to that shown in “**A**.” To note are the large lipid-like inclusions (arrowheads) in a dense-bodies (db) and the thin connecting tubule (arrow) with an adjacent, oval-shaped granule in the upper part of the micrograph. It is noticeable that the same axon shown in “**A**,” here (asterisk) reaches contiguity with the astrocytes plasma membrane. **(C)** A PAP contiguous to an axon (ax) whose myelin envelope protrudes (asterisk) to the surface of the former. To note are the disruption of that part of the myelin next to the dense-body in the upper part of the process (arrow). db, dense bodies; if, fascicle of intermediate filaments; m, mitochondria; rer, rough endoplasmic reticulum (rer). Specimen from the medial preoptic nucleus. **(D)** An example of a PAP bulging-out from an end foot to the neuropil of the ventro-medial hypothalamic nucleus. f, fascicles of intermediate filaments; G, Golgi apparatus; L, capillary lumen m, mitochondria. **(E)** A para-vascular astrocyte (AC) in the deep parietal cortex (i.e., Layer VIb). Note a proximal process that originates a PAP next to two myelinated axons (asterisk). **(F)** A large PAP (dotted) about a myelinated axon (boxed) in the olfactory bulb medulla. To be highlighted is the proximity between the process containing presumptive lysosomes and the myelinated fiber contiguous to it. **(G)** PAP (dotted) in the neuropil of the CA4-dentate intersection. Note the protrusion (arrow) and splitting of the myelin sheath encircling the axon next to the process. mf = mossy fiber. Calibration bars = 0.5 μm in **(A–D,F,G)**, and 2 μm in “**E**.” Adult rat brain.

**Figure 4:**
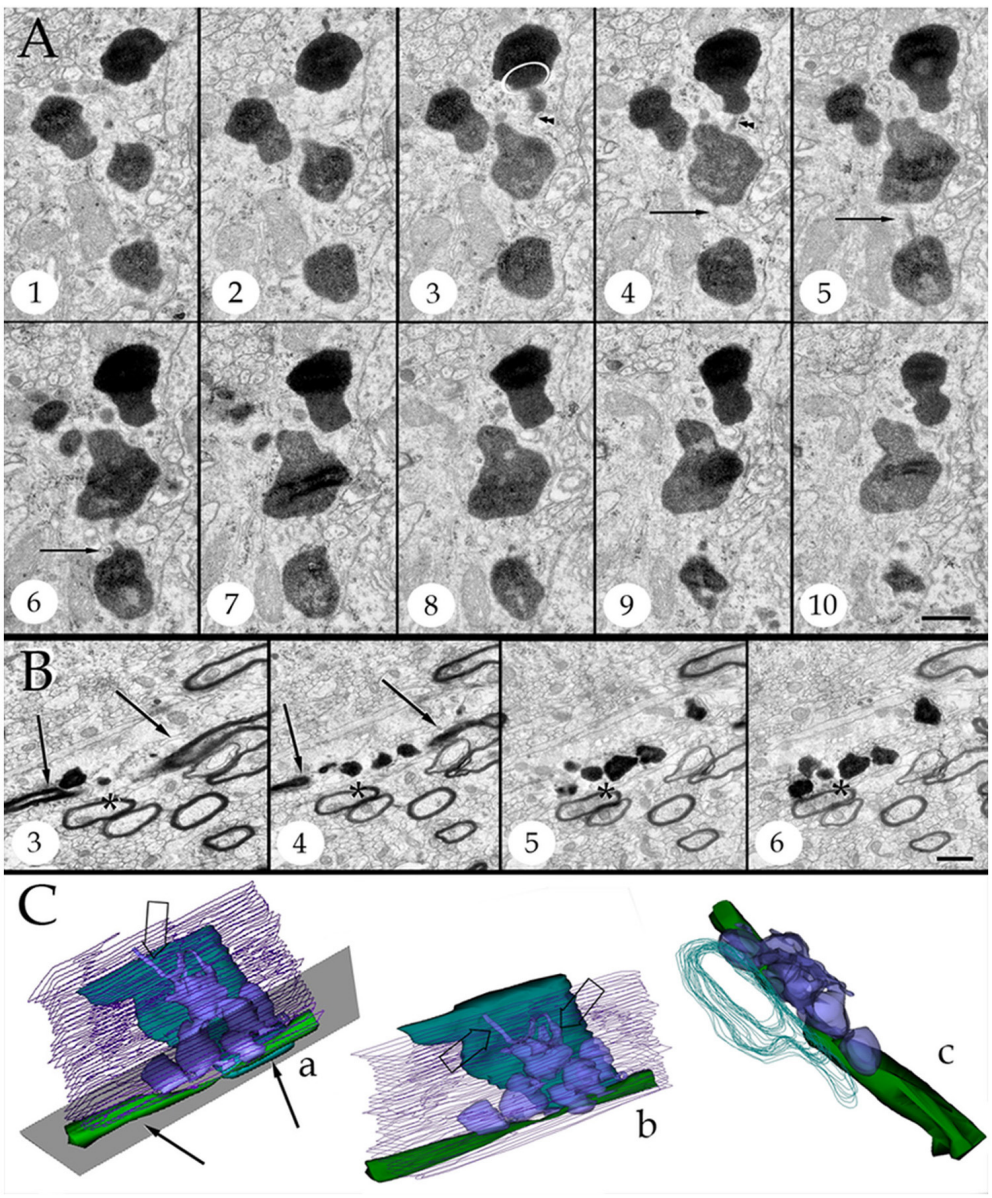
Series throughout paraxial processes. **(A)** Presumptive secondary lysosomes in the core of a paraxial process (PAP). To note are the side-to-side confluence (circle, section 3) or via thin communicating tubules (sections 3 and 4, arrowheads and sections 4–6, arrows). Deep frontal isocortex. **(B)** Series paralleling the myelinated fiber (arrows) in sections 3 and 4. To note is the progressive increase in both size and number of dense bodies overlaying the myelinated fiber. Asterisks label an axon reconstructed in “**C**” and coursing orthogonally throughout the series. Olfactory bulb medulla. **(C)** Reconstruction from 34 sections including those shown in “**B**.” **(a,b)** lateral views, showing the lysosomal (light blue) clustering and anastomoses within the PAP cytoplasm (blue). Next to the PAP myelinated axons course horizontally (green, arrows) and the orthogonally (turquois). Note the alignment of lysosomes with the myelin envelope and the short tubules (hollow arrows) anchoring in the plasma membrane (out-lined in blue) contiguous to the myelin covering. Gray = level of sectioning of micrographs shown in “**B**.” **(c)** Orthogonal view showing the group of lysosomes in apposition with the cross-sectioned axon (outlined in turquois). 3D figures were obtained with the “Reconstruct” software that is access-free (http://synapses.clm.utexas.edu/tools/reconstruct/reconstruct.stm). Adult rat, deep granule cell layer of the main olfactory bulb. Scale bars = 0.3 μm.

**Figure 5:**
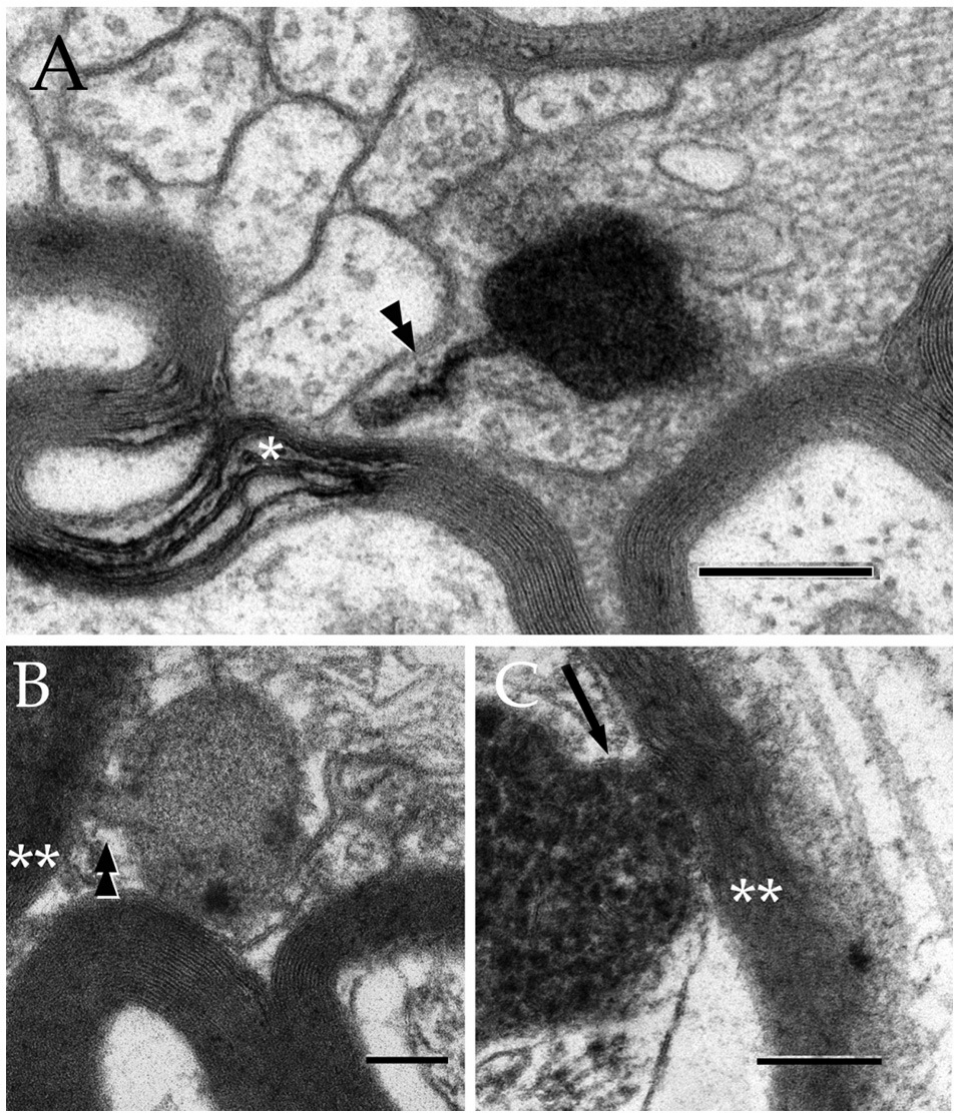
Secondary lysosomes (SL) in astrocytic processes. **(A)** A long, slender tubule running from the SL toward the process periphery. **(B)** A short tubule (arrow heads) bridging a SL to a distorted myelin envelope (double asterisk). **(C)** A SL fussed with the limiting plasma membrane of a paraxial process and opening to the altered myelin envelope (double arrow-head). To note is the continuity between the SL unitary membrane (arrow) and that of the process proper. Scale bars = 300 nm in **(A)**; 100 in **(B,C)**.

**Figure 6:**
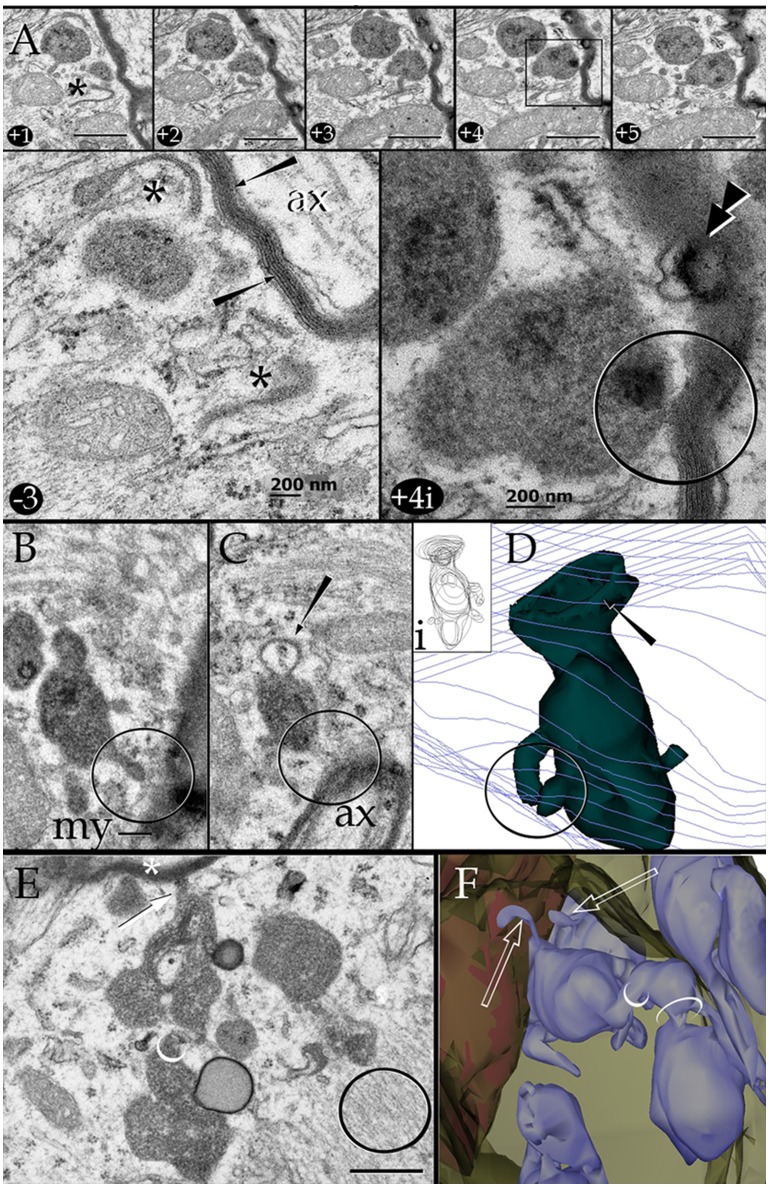
Series through the part of a paraxial process (PAP) in direct contact with a myelinated axon (upper right side). **(A)** As the sectioning proceeds throughout a lysosome, it opens (+4, black square and +4i, circle) to the PAP's surface, next to the myelin envelope. Observe that the myelin envelope collapses (+4i, circle) and embeds bubble-like formations (+4i, arrow heads) next to the lysosome opening. The myelin periodicity is restored in sections distal to the site of lysosome fusion (−3, arrows). Asterisks, inter-lysosomal loops. Adult rat, deep (i.e., layer VI) frontal isocortex. ax, axoplasm. **(B)** An electron-dense granule issuing a finger-like channel toward a distorted myelin envelope (my). **(C)** Alternate section, one section apart from that shown in “**B**,” showing the opening of the channel leading to an axon (ax) with a disrupted myelin envelope. Note the membranous appendage (arrow) attached to the upper aspect of the granule. **(D)** 3D reconstruction of the granule (green) sampled in “**B**” and “**C**.” It is evident that the “loop” shown in “**C**” is part of a funnel-like structure (arrow) apposing to the PAP's cell membrane (out-lined in blue). Circle, tubules shown in “**B**” and “**C**” opening to the process plasma membrane. **(i)**. Serial out-line of the granule in 21 successive sections. **(E)** Electron micrograph of a cluster of presumptive lysosomes in a PAP opening (arrow) to a myelinated fiber (asterisk) in the neuropil of the deep frontal cortex. The side-to-side communication (white circle) between them is evident. Black circle, bundle of intermediate filaments. **(F)** 3D from a series through the PAP shown in “**E**.” Note the lysosome clustering (pale blue) toward the myelinated fiber (red) and the narrow ducts (arrows) opening to the process cell membrane (pale green) contiguous to the myelin sheath. Circles, anastomoses between lysosomes. Scale bars = 0.25 μm in **(A)** +1 to **(A)** +5, **(B–F)**, and 200 nm in **(A)** −3 and **(A)** +4i.

**Figure 7:**
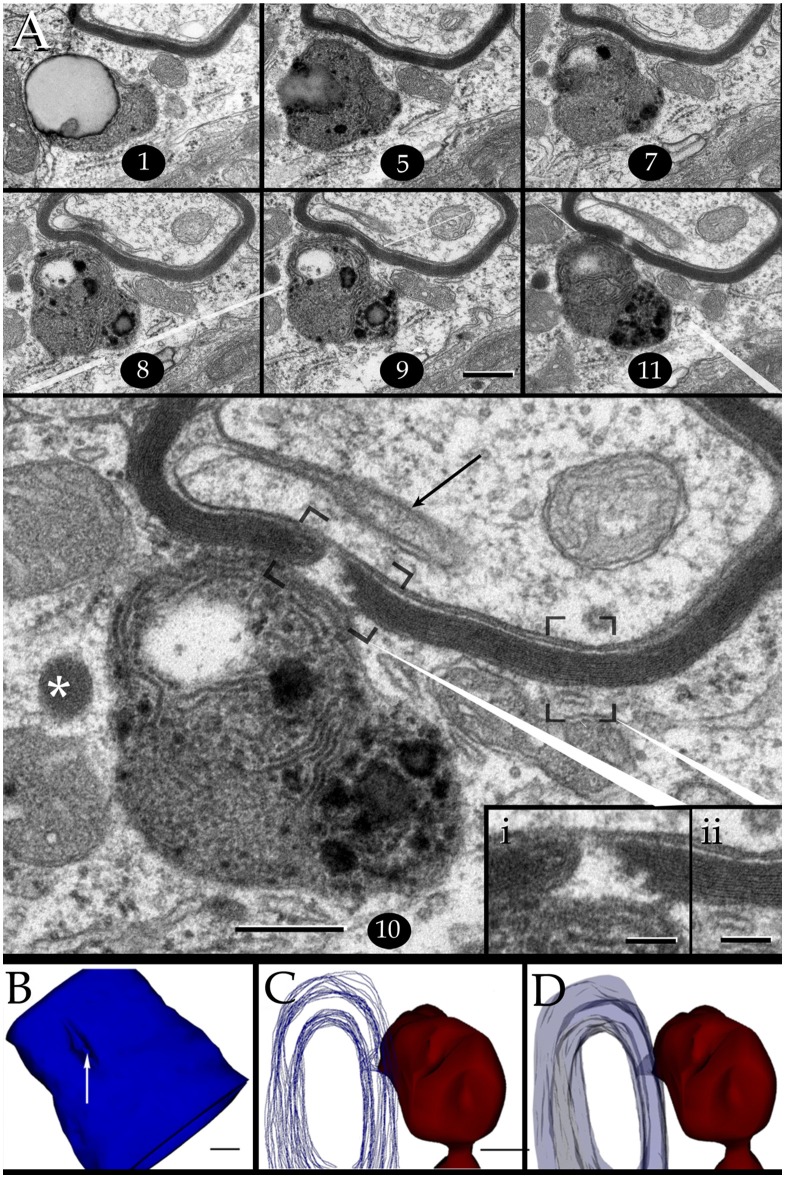
Interaction of lysosomes with an altered myelin envelope in the hippocampus. **(A)** Alternating (1, 5, and 7) and successive (8–11) micrographs through a series at the interaction of a paraxial process and the paranodal region of a myelinated axon. Dissolution of the myelin sheath is deeper in micrograph 10 (squared). Asterisk, presumptive primary lysosome. Arrow = oligodendrocyte tongue protruding to the axoplasm. **(i)** At higher magnification the focal excavation of the myelin envelope and the lysosome's membrane opening (right side) are evident. Also note the flocculent, electron-opaque, material embedding the myelin stumps. **(ii)** Myelin envelope just apart from the lysosome-myelin interaction displaying a regular, alternating, pattern. **(B)** 3D reconstruction showing the cavitation (arrow) of the myelin envelope shown in series depicted in “**A**.” **(C)** Note that the site of myelin (outlined in pale blue) indentation matches with that of the lysosome-(red) cell membrane fusion. **(D)** Ibid. Scale bars = 0.2 μm in **(A–D)**; 200 nm in inserts.

Survey views of the neuropil (Figure 3) reveal the presence of a group of medium-sized APs (i.e., 3–10 μm by 2–4 μm in the longest and transverse axes, respectively) that contain numerous electron-opaque granules, surrounded by free ribosomes, narrow cisterns of the rough endoplasmic reticulum, and two to four Golgi apparatus. Among these organelles, one or two distinct bundles of intermediate filaments course in assorted directions. These outgrowths are eccentric and, although they contain most organelles normally observed in the perikaryon (see, Larriva-Sahd, 2014), some differences are noticeable. The differences at first glance are the higher density, larger size, uneven profiles, and coalescence of the electron-dense granules in processes (Figures 3, 4A,B). Another characteristic common to these enlargements is their apposition to one or two myelinated axons coursing through the neighboring neuropil (Figures 3–**6**). Given that the organelles in these processes have a distinct asymmetrical distribution, protruding sidewise, and are common in the neuropil of most specimens utilized for conventional electron microscopy, they will be referred to as “para-axial processes” (PAPs) (Table 1). They are more frequent in samples from the olfactory bulb medulla (Figures 3A,B, 4B,C) and, in descending order of frequency, in the hippocampus (Figures 3G, **7**), piriform- (not shown), deep parietal- (Figure 3E), and frontal- (Figure 4A) isocortices; and the ventro-medial hypothalamic (Figure 3D), and medial preoptic nuclei (Figure 3C). Attempts to find PAPs in the deep cortical white matter or in optic nerves were unsuccessful. The foregoing description which derives from observations in single or assorted sections, precludes to ascribe the lysosomal nature of granules containing electron-opaque matrices as well as a possible specialization(s) of the PAP along its intersection with myelinated fibers. These methodological drawbacks are approached by combining histochemical and cytochemical techniques with 3D reconstructions from successive sections for electron microscopy as presented next.

**Figure 8:**
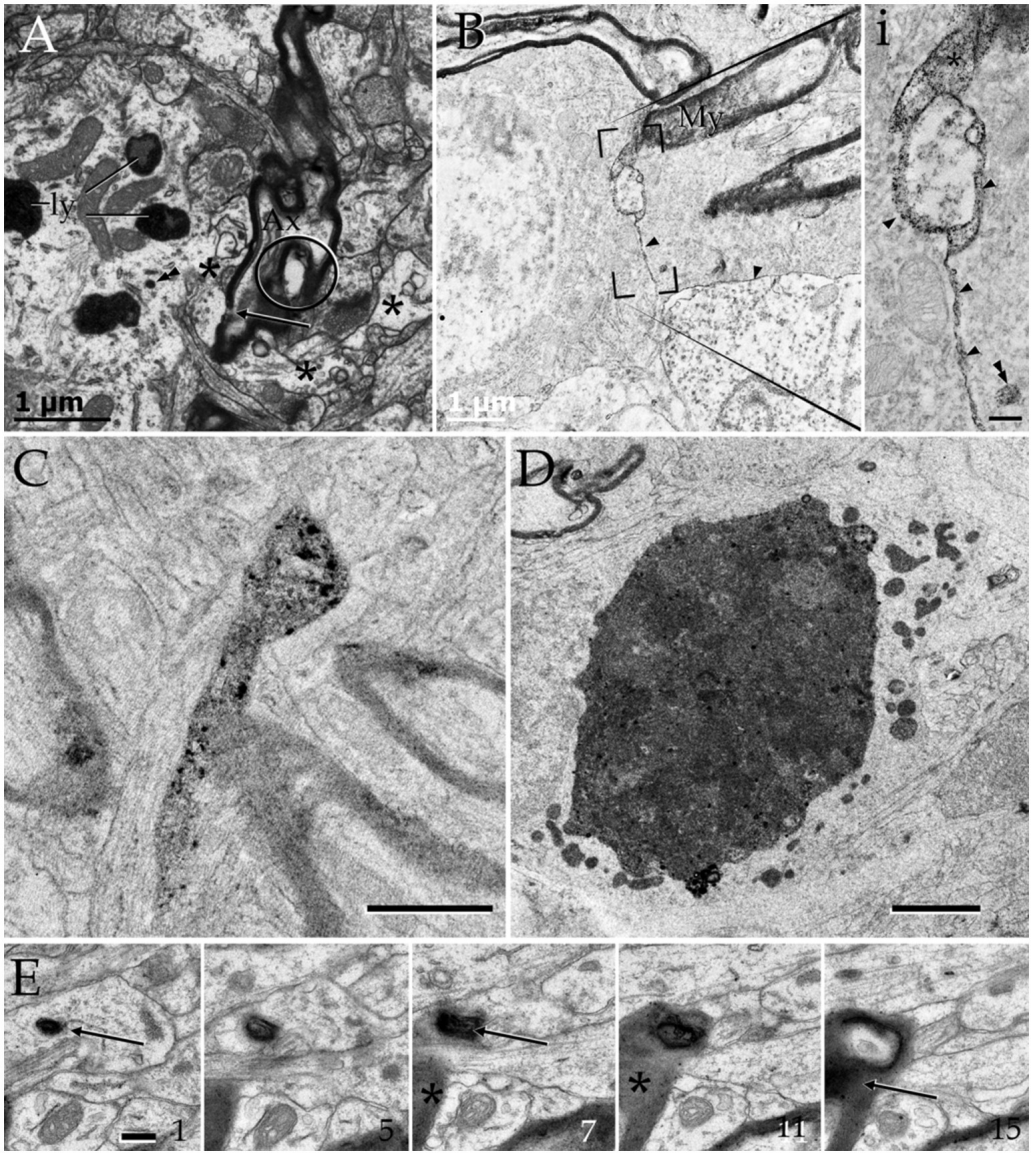
Cytochemistry to acid phosphatase in the neuropil of various forebrain areas. **(A)** A section through a paraxial process (PAP) (asterisk) counter-stained with uranium and lead. Note the electron-dense reaction product associated with lysosomes (ly) and a transverse interruption of the myelin envelope (arrow) at the PAP-axon intersection. Uranium-lead contrast. **(B)** Unstained specimen showing the site of membrane apposition of two astrocytes and a neuron (bottom-right). Note the inter-membrane reinforcement due to electron-dense reaction product. My, tangential section though the myelin envelope. **(i)** High magnification view at the site of convergence of the cell membranes and myelin sheath. Note the lead precipitate in the intercellular space (single arrowheads), myelin envelope (asterisk), and primary lysosome (double arrowhead). **(C)** Tangential section through a myelin envelope displaying dark precipitates to acid phosphatase. **(D)** A presumptive apoptotic cell surrounded by astrocytic processes with converging lysosomes. **(E)** Successive sections from an astrocytic process. Note the continuity of the reaction product to acid phosphatase from the core of the lysosome (arrow) to the adjacent myelin envelope (asterisk) in sections 7, 11, and 15. Scale bars = 1 μm in **(A–D)**; 200 nm in **(i)**, and (**E**).

**Figure 9:**
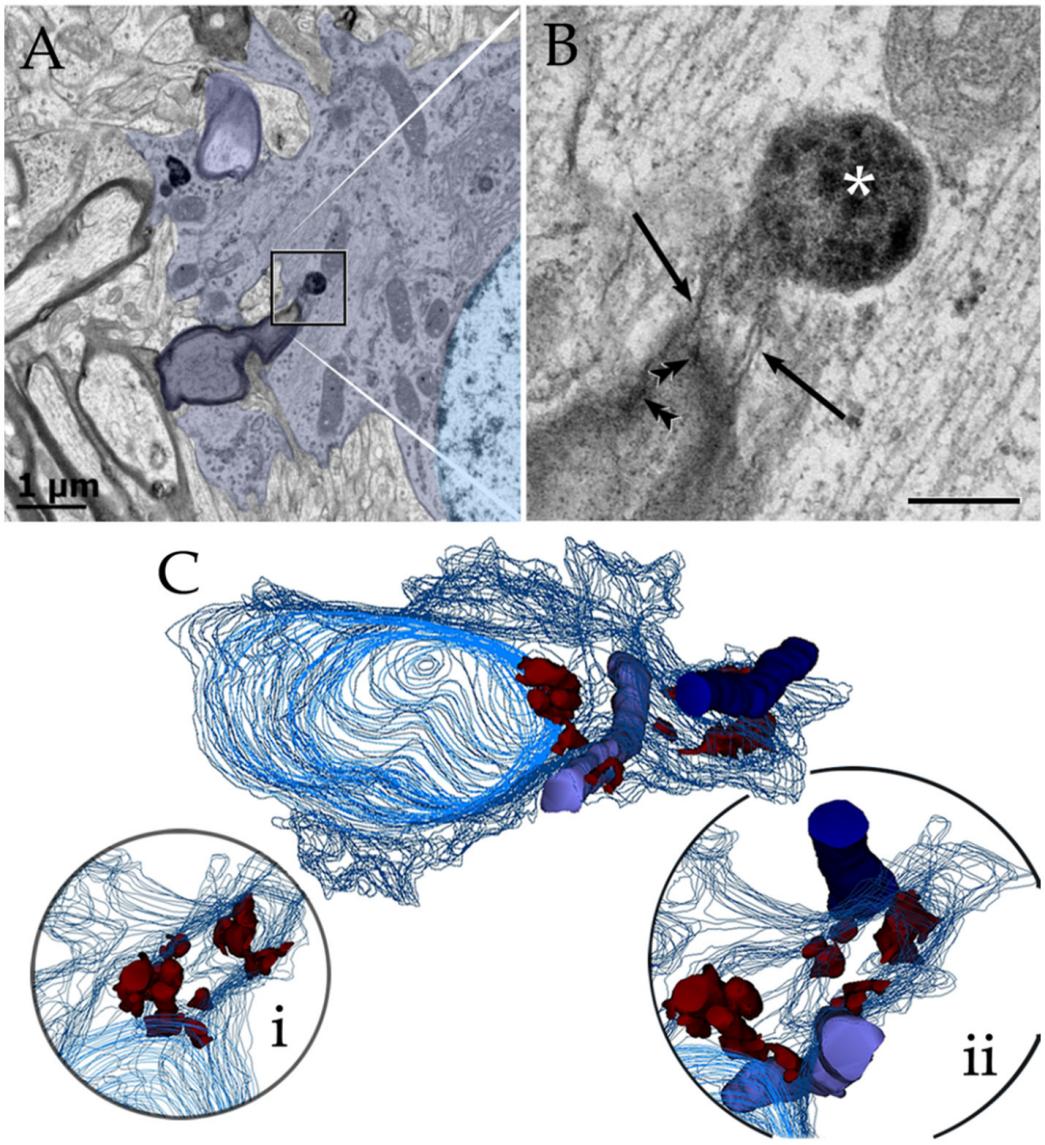
Astrocytic perikaryon and a paraxial process in the main olfactory bulb medulla following cytochemistry to acid phosphatase. **(A)** Survey view of a section through the perikaryon and a thick, paraxial process both containing lysosomes. **(B)** High magnification micrograph of the area framed in “**A**.” The electron-dense reaction product is associated with the lysosome matrix (asterisk) and focally with the myelin envelope (arrowheads). Note the narrow ducts (arrow) between the lysosome and the myelin envelope. **(C)** Reconstruction from all lysosomes found throughout the series. Note that lysosomes in the perikaryon and paraxial process are grouped in that part of the cytoplasm next to myelinated fibers (blue and light blue-colored cylinders). **(i)** Rotation of the model showing lysosomal polarization toward the plasma membrane (out-lined in blue). Cell nucleus, outlined in light blue. **(ii)** Myelinated fibers have been added to that image in “**i**.” From a series of 62 sections. Scale bars = 1 μm.

**Figure 10:**
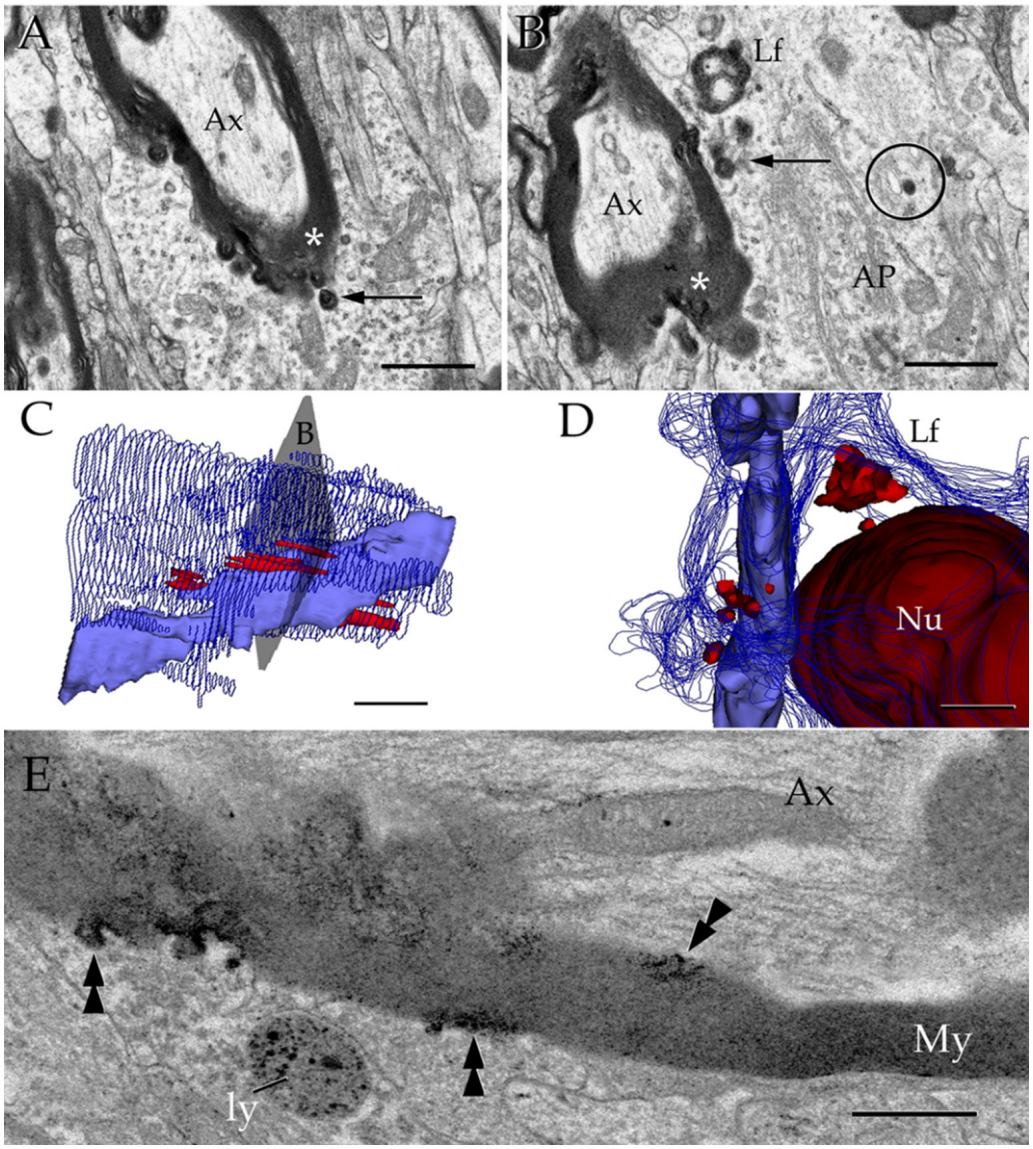
Interactions between the astrocyte and unusual axons with severe myelin disruption. **(A)** A process in the neuropil of the cerebral cortex is in opposition to a myelin sheath displaying marked disruption as evidenced for the uneven, beaded, profile, and focal substitution by an amorphous, electron-opaque material (asterisk). Ax, axoplasm. **(B)** Transverse section through an astrocytic process (AP) interacting with a disrupted myelin sheath due to infiltration by amorphous electron-dense material (asterisk). Arrows, presumptive phagosomes containing fragments of the myelin envelope. AP, Golgi apparatus; Lf, lipofuscin granule. Specimen from the olfactory bulb medulla. **(C,D)** 3-D views of the astrocyte shown in “**B**” (gray). All lysosomes (bright red), and a lipofuscin granule (Lf) have been reconstructed. To note is the cluster of lysosomes in the part of the karyoplasm next to an uneven axon with a distorted myelin sheath (pale blue). Deep blue, astrocytic cell membrane; deep red, cell nucleus. **(E)** Electron micrograph of a specimen from the olfactory bulb medulla incubated for acid phosphatase visualization. The high magnification micrograph allows visualization of the site of intersection between a paraxial process (bottom) and the altered myelin envelope (My) of the axon (Ax). Note that the electron-opaque reaction product concentrates at sites where the myelin envelope losses its periodicity (arrow-heads) and is substituted by amorphous material, as well as in the matrix of a lysosome (ly). My = myelin envelope with preserved membrane periodicity. Scale Bars 0.5 μm in **(A–D)**; 0.2 in **(E)**.

Presumptive primary lysosomes (PLs) are small (170–210 nm in diameter), rounded granules that contain a homogeneous matrix of moderated electron-density. In the PAP PLs usually lie next to the trans region of the Golgi apparatus (Figures 3B, **10B**; Essner and Novikoff, 1962; Novikoff, 1963; Nichols et al., 1971; Fawcet, 1981). Secondary lysosomes (SLs; Figures 3–**10**) are the most distinctive organelles of the PAP. SLs are larger (<0.5 μm) and barely oval due to large, convex excrescences that give them an overall overinflated appearance. SL content is generally electron-dense, but it varies from one SL to the next (Figures 2, 4A). The granular matrix fills up the entire granule; however, lipid droplets and/or membranous debris, are not infrequent, approximating 3 out of 10 SLs (Figures 3B, **7A**). While the presence of lipid-like and membrane inclusions is a feature that SLs (see Fawcet, 1981) and lipofuscin granules (see (Peters et al., 1976; Saprunova et al., 2010)) have in common, the larger size, pleomorphic shape, and contents of the latter (**Figures 10B–D**) allow identification. A membranous appendage is observed at the surface of about a third of SLs, which consists of a loop made up of a thin duct (i.e., 30 nm in width) outlining a circular area of 0.3 μm in diameter (**Figure 6A**, sections +1, −3; **Figure 6C**). Serial sections through these “loops” reveal that they may correspond to a conical, funnel-like compartment that occasionally anchors with the PAP cell membrane (**Figure 6D**).

### Secondary lysosomes anastomose and open to the plasma membrane

SLs coalesce with one another via latero-lateral anastomoses (Figures 3B, 4A,C) and/or via tiny tubules of varying diameters (Figures 4A,C). The latter consists of a finger-like duct whose lumen may be tubular (30 nm) or uneven (30–100 nm) in width and have also a variable length (i.e., 0.04–0.3 μm). Observation of series makes evident that SLs located next to the part of the PAP that interact with a myelinated fiber (see below) open to the limiting plasma membrane through one or two of the narrow ducts just described (Figures 4C, 5A,B, 6B,C, **9B**) or by direct fusion of the SL with the plasma membrane (Figures 5C, **8E**). Summarizing, SL clusters structure a membranous, communicating system that opens up to the PAP vicinity, usually next to one or two myelinated fibers.

### Lysosmes and disrupted myelin exhibit reactivity to acid phosphatase

When beta-glycerophosphate is used as the substrate, both PLs and SLs exhibit an electrondense precipitate that may underscore the granule membrane or be diffuse, thus confirming their lysosomal nature (DeDuve, 1983; **Figures 8**, **9A,B**, **10E**). Additionally, the reaction product is associated with AC's nuclear envelope and with the Golgi apparatus (see Essner and Novikoff, 1962; Novikoff, 1963; Goldfisher et al., 1964; not shown). Focal electron-dense deposits are common in the myelin envelope of certain axons (**Figures 8B–D**, **10E**). Serial sections through areas of lysosome-myelin interface reveal continuity of reactive deposits bridging them (**Figure 8E**). Lysosomes in APs surrounding pyknotic cells exhibit both intense reaction products (**Figure 8D**). Finally, like for the PAP-myelin intersections, the reaction product for acid phosphatase can be seen within the extracellular aspect of the plasma membrane bounding astrocytes and neurons (**Figures 8B,Bi**).

### Lysosomes in astroglial processes interact with altered myelin envelopes

Axons in the vicinity of the PAP vary in dimensions as a function of brain area (Table 1), and they have a mean transverse diameter of 0.98 μm. Most axoplasms (i.e., 85%) contained the usual, undisrupted organelles, without structural evidence of damage or improper fixation (Peters, 1970). However, varying degrees of internodal myelin disruption are found in axons interacting with the PAP. Areas of myelin collapse are well circumscribed and measure between 0.08 and 1.5 μm in length. Furthermore, they present two basic structural patterns. The most common pattern consists of focal substitution of both intraperiod and major dense lines by amorphous and electron-dense material (Figure 7A, section 10). Embedded in these areas of rarefaction, pleomorphic, and donut-like figures of electron-opaque material may be seen (Figure 6A, sections +4 and +4i). Observations of areas of the PAP-altered myelin interphase reveal that they coexist with the part of the former where SLs coalesce with the cell membrane; series in Figure 6 illustrates the site where an SL fuses with the PAP unitary membrane and the aforementioned myelin alterations arise (Figure 6A, sections +4 and +4i); however, the myelin envelope that is located separately or in distal sections from the myelin rarefaction, acquires the distinct, alternating pattern, observed in normal myelin (Figure 6A, sections +1 and −3; Figure 7A, sections 1, 5, and 8). A second alteration of the myelin envelope contiguous to a PAP, which is observed in one third of the series, consists of a transverse interruption of the internodal myelin sheath (Figure 7, panels 9–11; Figure 8A, arrow). As shown in Figure 7, transmural transverse dissolution of the myelin leaving denuded areas of the axolemma defines this sort of alteration. The myelin stumps at either side of the gap display rarefaction in a similar fashion to the one just described for the previous alteration. Beyond this focal area of myelin disruption, again, the structure of the normal myelin is observed (Figure 7, insets). Series throughout areas of myelin disappearance reveals that they may be conical or bowl-shaped and coexists with that part of the PAP where SLs appose or lead to its plasma membrane (Figure 7A, section 10, and Figures 7B–D).

Myelinated fibers displaying severe internodal disruption are occasionally observed [i.e., 10 times in the olfactory bulb (**Figure 10B**), three in the cerebral cortex (**Figure 10A**), and one in the ventromedial hypothalamic nucleus (no shown)]. Internodal myelin of these relative infrequent axons show widespread tumefaction, dissolution, and “ballooning” (Peters, 2009a,b; Figure 8A, circle). Phagocytosis of presumptive fragmented myelin next to these severely damaged axons (**Figures 10A,B**) mimics that observed in senescence (Peters, 2009a) or in advanced stages of experimental axonal transection (Nathaniel and Pease, 1963; Franson and Ronnevi, 1984). Reconstructions throughout PAPs and AC perikarya display both progressive increase in lysosomal number and occurrence contiguous to the disrupted myelin envelope (Figures 9C, 10C,D).

### Lysosome high incidence and fusion occur in lysis but absent in glomeruli and tripartite synapses

**Figure 11:**
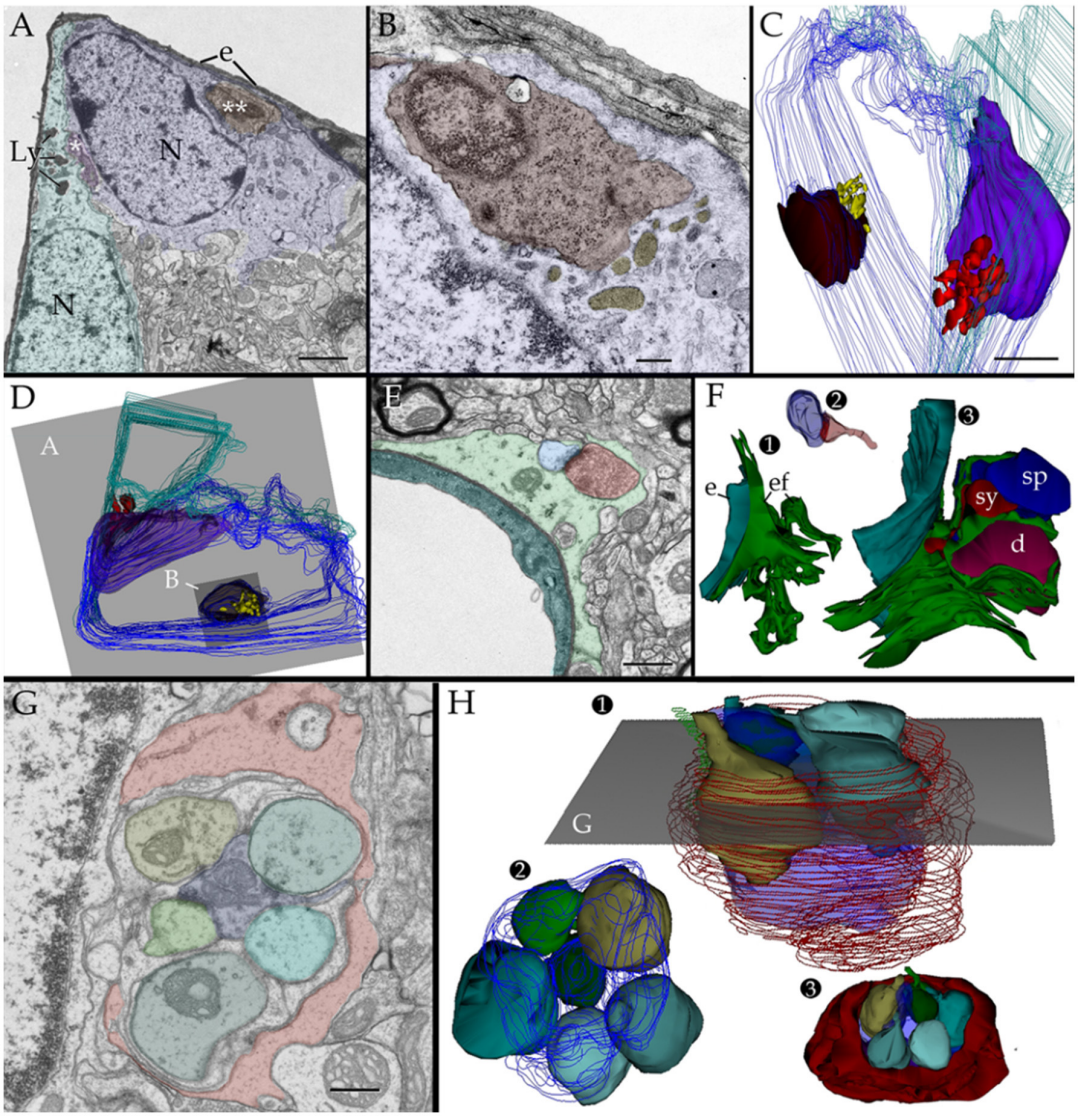
Two and three dimensional views of the astrocyte and its processes in the neuropil. **(A)** Micrograph at the site of ramification of two capillary blood vessels that arbors two astrocytes (green and blue). Between the astrocyte's cell body (light blue) and the endothelial cell (e), a pyknotic cell (double asterisk) can be seen. Another dark cell (single asterisk) is identified between the two astrocytes. Note the clustering of electron-opaque granules (Ly) in the cytoplasm adjacent to the latter dark cell. **(B)** High magnification view from a successive section. The presumptive apoptotic cell (light purple) is surrounded by the outer aspect and a thin process of the astrocyte. To note are the frequent electron-dense bodies (light yellow) in the part of the cytoplasm next to the apoptotic cell. **(C)** 3D view of all the electron-dense granules (red and yellow) contained by both astrocytes within the series. Note that all of them are clustered in the area of the cytoplasm adjacent to pyknotic cells (brown and purple). **(D)** Survey 3D view depicting the levels of sectioning. **(E)** Electron micrograph of the capillary wall and neuropil in the ventro-medial hypothalamic nucleus. A “tripartite” axo (red)-spinous (blue) synapse is shown. Note is that the synaptic terminal is covered by a glial laminae from the end-foot (green) devoid of electron-dense granules. **(F)** 3D views of the end-foot and synapse shown in “**E**.” **(F1**) End-foot (green) and endothelium (turquois). **(F2)** 3D model of the axo-spinous terminal. **(F3)** Position of the synaptic terminal encased by a glial envelope. **(G)** Electron micrograph of a glomerulus in the granule cell layer of the olfactory bulb. Note the concentric arrangement of the astrocytic lamella with a paucity of organelles. **(H1)** Dendritic spines (solid colors) receiving synaptic contacts from the single bouton at the center (transparent blue). **(H2)** Rotation of the 3D model to depict the six dendritic spines tributary to a centrally located bouton (outlined in blue). **(H3)** External appearance of the glial covering of the glomerulus. Note that glial processes (red) encase the glomerular neural elements. Scale bars = 1 μm in **(A–D)**; 0.5 in **(E–H)**.

If observations regarding localization, fusion, and acid phosphatase positivity of the PAP counterpart with a lytic process, they may be mimicked by other known lytic phenomena (Tasdemir-Yilmaz and Freeman, 2014) or be absent in APs normally associated with synapses. This is tested first in the olfactory bulb by observation of phagocytosis of apoptotic neuroblasts by the AC (He et al., 2006). In sections incubated with beta-glycerolphosphate lysosomes gather and fuse with the plasma membrane and both the lysosomes and the shrunken, presumptively apoptotic cell, display positivity to acid phosphatases (Figures 2D and 8D). To define if this takes place within a phagosome or, extracellularly between the AC and its processes, observation of series throughout the AC-shrunken cell(s) intersection was performed. Figure 11 reproduces an example from two pyknotic cells trapped between two ACs next to the capillary wall. Reconstruction of all electron-dense granules in the AC's cytoplasm throughout the series (Figures 11C,D), reveals that lysosomes lie, and some of them coalesce, with the plasma membrane surrounding pyknotic cells, thereby interacting directly with that of the disrupted cell in the extracellular domain.

Additional series through tripartite synapses (Figures 11E,F; Araque et al., 1999; Ventura and Harris, 1999; Witcher et al., 2010) and glomeruli (Figures 11G,H; Price and Powell, 1970b; Peters et al., 1976) in the olfactory bulb medulla confirm their distinct 3D and inner structure as defined elsewhere; although, in our sample tripartite synapses may occasionally be seen entangled by the AC's end-foot (Figures 11E,F). APs surrounding axodendritic terminals or glomerular complexes are devoid of electron-dense granules as defined by 3D reconstructions (Figures 11F–H).

### Oligodendrocytes and microglia

**Figure 12:**
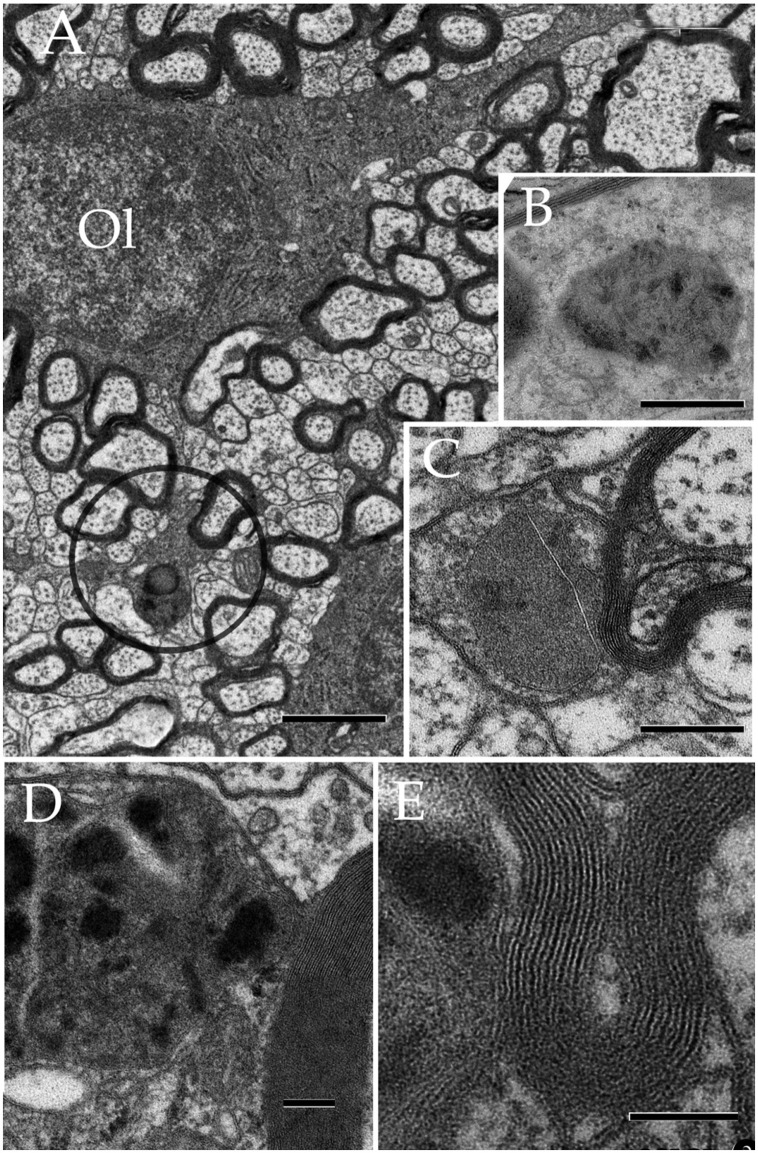
Oligodendrocyte and its processes in the neuropil. **(A)** Oligodendrocyte (Ol) with divergent processes piercing the neighboring neuropil and numerous myelinated axons. To note is the drum-stick appearance of the descending process that is crowned by a round, terminal (circle) (RTK) knob containing a large, solitary dense-body. Olfactory bulb medulla. **(B)** A lysosome in an Ol process displaying activity to acid phosphatase (dark, electronopaque deposits). **(C)** An RTK containing a putative lysosome in apposition with the process plasma membrane and, the later with a normal-appearing myelinated fiber. Cerebral cortex. **(D)** Higher magnification view of an RTK following incubation with a substratum containing B-glycerol phosphate. To note are the electron opaque products of the reaction and the continuity of the lysosomal membrane next to an adjacent normal-appearing myelin envelope. Specimen from the ventral lateral nucleus of the hypothalamus. **(E)** A thinner. i.e., 60 nm, section displaying the continuity of the RT plasma membrane, paralleling the outer aspect of the normal-appearing myelin sheath next to it. D and E specimen the CA4 sector of the hippocampus. Scale bars = 1 μm in **(A)**, 200 nm in **(B,C)**, 100 nm in **(D,E)**.

Observations of oligodendrocytes (Ols) in the neuropil of assorted specimens, depict the well-known interaction of this cell type with the intact myelin envelope (Peters et al., 1976). As shown in panel Figure 12, the Ol issues two or more processes that course radially in intimate apposition to intact myelinated axons. It is noted that some Ol processes resolve into distinct rounded structures, termed here round terminal knobs (RTK). A RTK contains a likewise rounded, solitary, granule that contains a pleomorphic matrix. The latter measures 0.3–0.7 μm in diameter and it contains lipid-droplets, membranous, and/or cotton-like inclusions. Granular contents are embedded in a homogeneous matrix of a moderate electron-density. When incubated with the appropriate medium to acid phosphatases (see above) the RTK granule exhibits focal positivity due to electron-opaque deposits (Figure 12B). Bias observation of 250 RTKs, depict that most of them (87%) appose to the myelin envelope of axons in the RTK's vicinity (Figures 12C–E). These axons are invariably invested by an intact myelin envelope. Although, the solitary lysosome of the RTK frequently interacts with the limiting plasma membrane and this with the myelin envelope proper, no fusion or continuity or enzyme reactivity were noted between the former and the latter.

**Figure 13:**
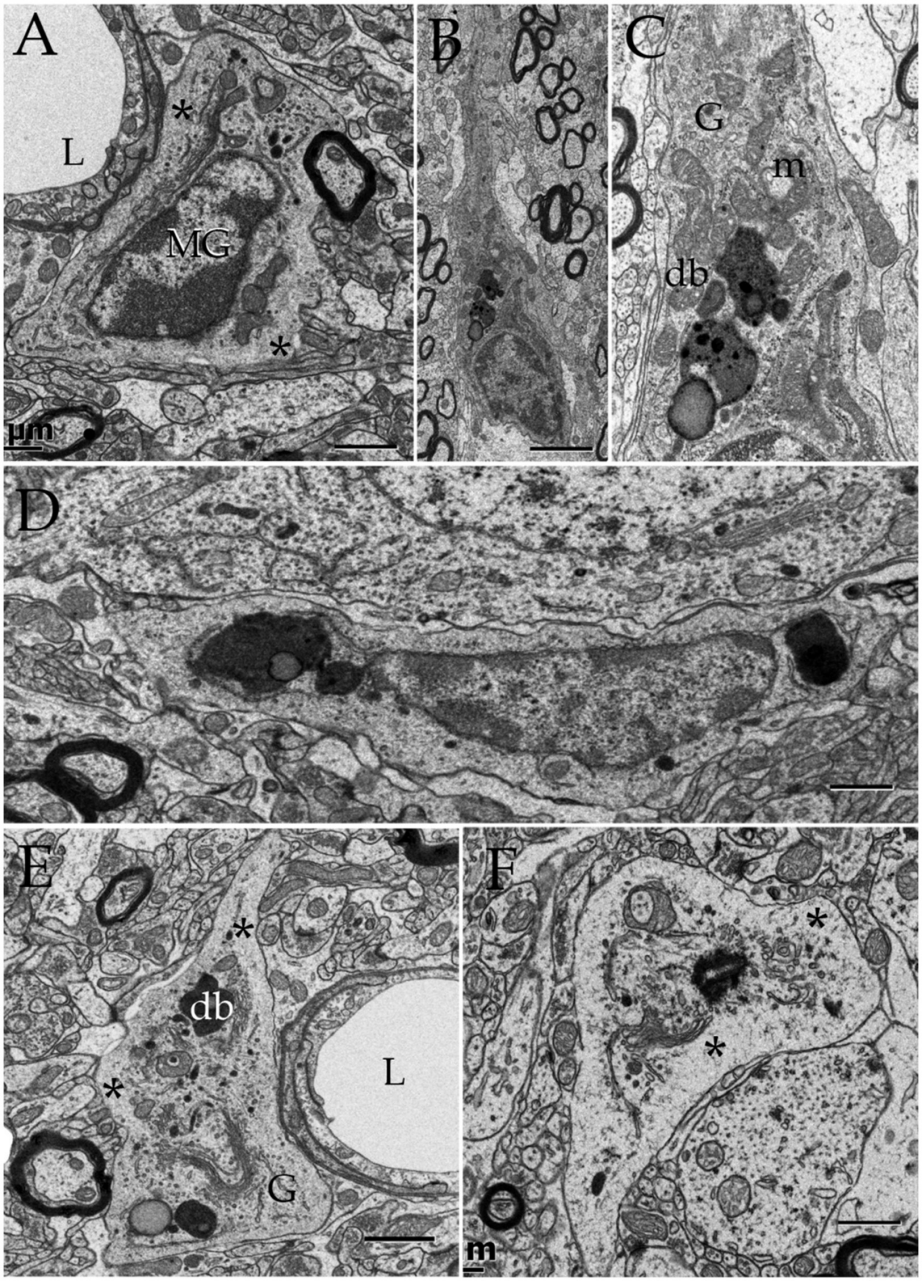
Microglia (MG) and its interactions with the neuropil in the normal neuropil. **(A)** Paravascular microglia (MG) laying between the capillary basal lamina and neuropil (right bottom). To note are the heterochromatic clumps outlying the nuclear envelope and the concentration of organelles within the perinuclear domain, thereby leaving an organellefree, peripheral cytoplasm (asterisks). L, capillary lumen. Deep temporal cerebral isocortex. **(B)** A slender MG interacting with the neighboring neuropil. To note is the distribution of electron-dense granules and other organelles within the core of the perikaryon and the ascending process. **(C)** Higher magnification view depicting the organelle types and distribution in the juxtanuclear area. Db, electron-dense granules; G, Golgi apparatus; m, mitochondria. Ibid. **(D)** Spindle-shaped MG lying next to a neuron (upper half) and neuropil (bottom). Deep frontal isocortex. **(E)** MG process, presumptively proximal, harboring similar organelles than those in the perikaryal domain (see “**A**,” “**C**,” and “**D**”) leaving an organelle-free halo (asterisks). db, electron-dense granules; G, Golgi apparatus. **(F)** MG process in the neuropil of the medial preoptic nucleus. Ibid; asterisks, organelle-free halo (asterisks). Scale bars = 1 μm in **(A–D)**; 0.3 in **(E,F)**.

Lastly, microglial cells (MGs) are relatively infrequent throughout the neuropil (see Peters et al., 1976). From the thirty six MGs perikarya identified here, most of them lie next to the capillary wall (Figures 13A,E); whereas a low proportion of them is encased by the neuropil (Figures 13B,C,E,F) or, rarely, in direct apposition to neurons (Figure 13D). Generally, MGs do not interact directly with myelinated axons and, if so, envelopes are well-preserved. To emphasize is that, like the MG's cell body, organelles in distal processes are surrounded by an organelle-free area cytoplasm underscoring the cell membrane (Figures 13E,F). Interaction of the MG's presumptive lysosomes with the cell membrane is virtually absent in the normal neuropil.

### Morphometry

Myelinated axons, PAPs, and RKTs numerical densities are summarized in Table 2.

**Table 2:** Numeric density of myelinated axons, astrocytic paraxial, and oligodendroglial processes in 70,000 μm^3^ of tissue.

	**Temporal isocortex**	**Olfactory bulb**	**VMHN**
Number of axons	1,854	9,050	1,600
Number of PAPs	5	23	6
Number of RTKs	2	5	2
PAPs/myelinated axons	1:370	1:393	2:266
RTKs/myelinated axons	1:927	1:1810	2:800

*VMHN, Ventromedial hypothalamic nucleus; PAP, Astrocytic paraxial process; RTK, Oligodendrocyte round terminal knob*.

## Discussion

**Figure 14:**
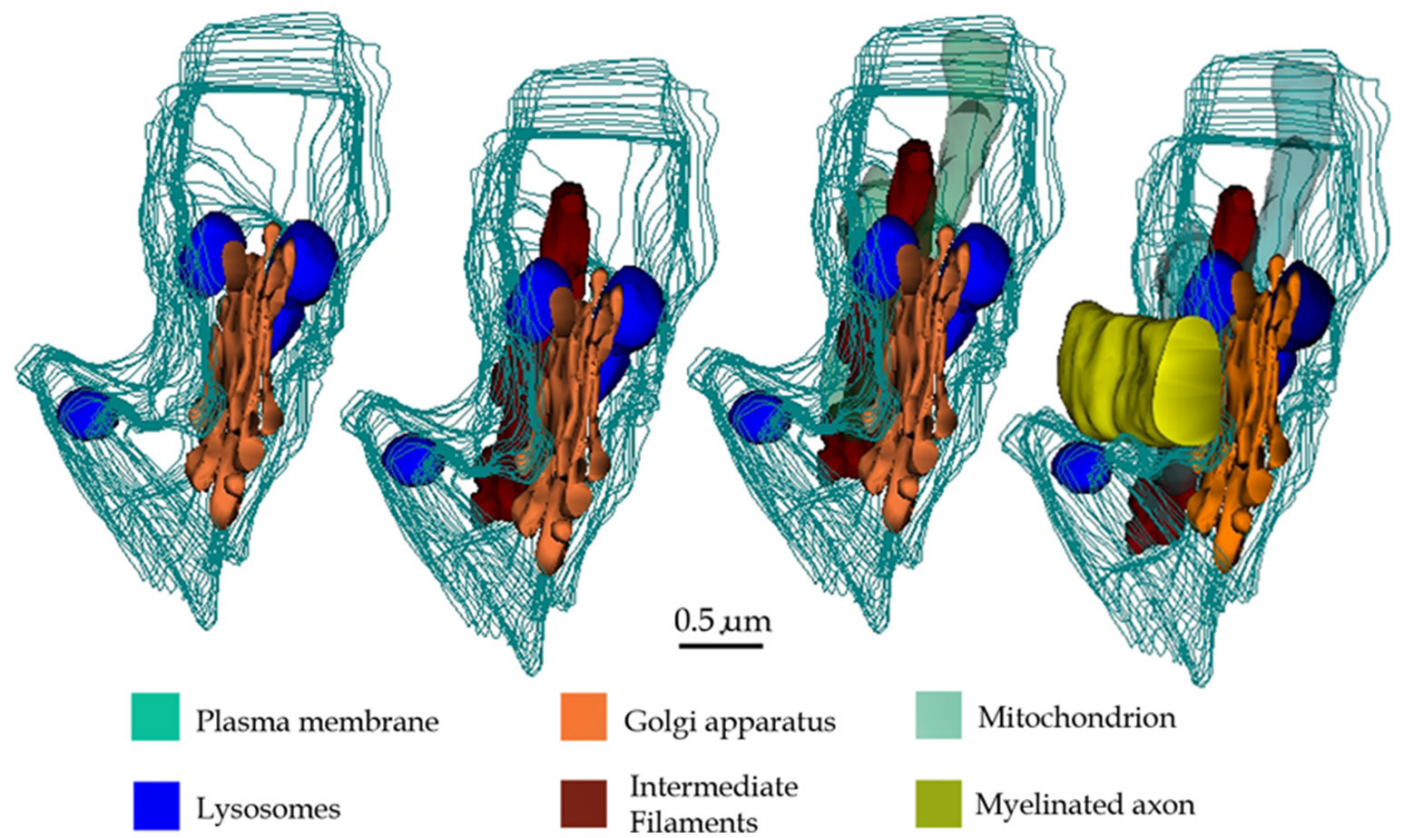
3D reconstructions showing organelles contained by a typical paraxial process (PAP) (out-lined in turquois). Progression (from left to right) of the reconstruction adding the PAP organelles and an adjacent myelinated axon to the succession.

Early light microscopists assumed that small clusters of myelin-like material (i.e., “myelinoid bodies”) in the white matter might represent sites of metabolic myelin turnover (Elzholtz, 1898; Jacob, 1912). This notion was strengthen with the advent of the electron microscope. To the best of our knowledge, two pioneer studies (Hildebrand and Skoglund, 1971; Hildebrand, 1977) in normal animals provide cytological evidence for the interaction of glial processes and myelinoid bodies. From these studies it was clear that glial cells internalize disrupted myelin figures. Hildebrand (1977) concluded that: “if these bodies represent a morphological expression of a myelin turnover, the oligodendroglial cell should be the main element responsible for their breakdown,” adding: “in the rabbit and Guinea pig myelinoid bodies are also present in the astrocytic cytoplasm.” Indeed, the interaction of lysosomes in the astrocyte's perikaryon with myelinoid bodies was illustrated for the first time (33a and 33b, Hildebrand and Skoglund, 1971). Recent work in adult frogs (Mills et al., 2015), has demonstrated the direct involvement of ACs in myelin remodeling during metamorphosis. Present observations support the belief that AC in the normal rat may normally be involved in myelin modeling. In fact, a group of astrocytic processes (APs) was identified in a variety of random specimens of the forebrain of healthy adult rodents. Due to the distinct structure, high incidence, and ubiquity, of this process, it was named “paraxial process” (PAP). PAP is defined as a differentiated collection of secretory organelles protruding from the AC process, which interacts with the disrupted myelin envelope of nearby axons (Figure 14). Distinct clusters of lysosomes linked both among themselves and with the limiting cell membrane, surrounded by secretory organelles allow PAP identification. With confocal microscopy performed on specimens from the mouse model expressing fluorescence to glial fibrilar acidic protein (eGFP), presumptive PAPs exhibited co-labeling to myelin basic protein, and lysosomes (Lyso-Tracker). While a PAP was readily identified in specimens intended for routine electron microscopy, the chances of visualizing the full PAP structure were diminished due to the drawback of observing assorted sections. In series, the presence of clusters of confluent SLs proved to be an effective landmark for PAP identification, as revealed by further inspection of the consecutive sections. This approach enabled PAP identification throughout most brain areas (Table 1) suggesting a ubiquitous distribution. The PAP appears to correspond to the normal counterpart of further differentiated glial processes induced by experimental crushing (Hildebrand et al., 1994) or immune (Gotch and Löhler, 1990; Ponath et al., 2017) damage to myelinated axons. While lysosomal activity in the PAP appears to be recruited in homeostatic myelin modeling, at the present, descriptive stage, confirmatory functional studies (see Tasdemir-Yilmaz and Freeman, 2014; Ponath et al., 2017; Zorec et al., 2017) are required to define that the lysosomal contents released in the myelin envelope cannot be deleterious and cause myelin disruption itself. So far, our contention in favor of the direct involvement of the AC in myelin plasticity along the lifespan is relevant in that myelin clearance throughout adulthood has customarily been ascribed to microglia (see Safaiyan et al., 2015) and macrophages in both the normal and the diseased central nervous system (see Nave and Werner, 2014), respectively.

The vesicular contents and the common association of the PAP with the disrupted myelin envelope of contiguous axons (Figure 14) suggests that the PAP represents an active site of myelin turnover. A PAP contains the necessary organelles for synthesis and exportation of secretory products as may be suggested from the previous observation that ACs express genes associated with cell secretion (Cahoy et al., 2008, vide infra). In the supramolecular domain, SLs in the PAP sculpt confluent units, as each one of them is interconnected with its respective homolog which in turn opens to the processes surface next to the altered myelin envelope. The reaction product to acid phosphatase within the SLs and membranes of the altered myelin, coupled with the focal reactivity observed in the intercellular space, provide complementary evidence for a lysosome-mediated myelin-lysis (see Tasdemir-Yilmaz and Freeman, 2014). While in neurons lysosomes are directly involved in extracellular dendritic spine plasticity (Chung et al., 2014; Padamsey et al., 2017), the release of lysosomal contents by ACs into the extracellular compartment, degrading materials therein, is uncommon for the normal nervous system. Previous observations of the normal, specialized, connective tissue recapitulate this process. In effect, the osteoclast, a large multinucleated cell of the normal bone tissue, lies in direct apposition to the bone surfaces exposed to the periosteum or endosteum (Ham, 1969). Upon systemic endocrine requirement, growth, or trauma, osteoclasts concur to lysate the bone matrix to be removed. The success of this process relies on the massive exocytosis of the lysosomal hydrolases next to the exposed bone matrix (Göthlin and Ericson, 1971; Matsuda, 1992; Zaidi et al., 1993; Szewczyk et al., 2013). Furthermore, lysosome occurrence, location, and fusion in our control series throughout the AC involved in engulfing presumptive apoptotic cells (Figures 11A–D; Nixon and Cataldo, 1993; Schmechel, 1999; He et al., 2006), replicate those observed for the PAP. On the other hand, astrocytic processes surrounding tripartite synapses (Figures 11E,F) and glomeruli (Figures 11G,H; Price and Powell, 1970b), both of which are functional constituents of the neuropil, no electron-dense granules are observed throughout the APs. Thus, the use of appropriate positive and negative inner controls of lysis coupled with the well-known mechanisms of intercellular matrix turnover, suggests that the PAP may normally be involved in the AC myelin remodeling.

Regarding the structural alterations of the myelin envelope of axons adjacent to presumptive sites of lysosome secretion, present and previous observations support that they correspond to sites of myelin collapse. The fact that myelin splitting may be an artifact of fixation and plastic embedment (see Peters, 1970; Möbius et al., 2016), led us to adopt the conservative criteria of considering fusion and dissolution of the myelin membranes as indicative of myelin damage (Nathaniel and Pease, 1963; Hildebrand and Aldskogius, 1976; Franson and Ronnevi, 1984; Pineas et al., 1984; Hildebrand et al., 1994; Dupree and Popko, 1999; Han et al., 2008; Peters, 2009a,b). Axon measurements and quantification (Tables 1, 2) disclosed that those associated with PAP are the most numerous throughout the neuropil (not shown). Since physiological properties of axons correlate with their diameter (Jack, 1975) we speculate that myelin-disruption compromises sets of axons associated with distinct functional task(s).

While evidences provided here and those gathered from prior work speak in favor of a lytic involvement of the PAP (see above), the possibility that other secretory product(s) ascribed to the AC are released though a lysosomal route remains to be defined. First of all, acid hydrolases represent only one-half of the total protein content of the lysosome (Softing and Klumperman, 2009), suggesting that at least some non-enzymatic products may also be secreted (Cotrina et al., 2000; Zhang et al., 2003) by this route. Second, although of numerous transmitter, trophic, homeostatic, and immunological secretory products are released by the AC (Sperri et al., 1997; Sobue et al., 1999; see Cahoy et al., 2008; Tavaggia et al., 2010; Lundgaard et al., 2014; Nave and Werner, 2014; Anderson et al., 2016), the involvement of lysosomes in this process has also been recognized. For instance, direct evidence by Zhang et al. (2003, 2007) supports that the ATP, a nucleotide with modulatory effect that is released by the AC (Queiróz et al., 1997), utilizes the lysosome as its secretory pathway (Cotrina et al., 2000). Furthermore, recent work (Padamsey et al., 2017) in pyramidal neurons puts forth the secretory pathway of lysosomes in dendritic spine plasticity. Thus, the possible involvement of PAPs as a secretory substratum should be left as an open, though testable, issue.

As expected, the number of myelinated axons varied as a function of site, being more numerous in the olfactory bulb medulla and comparable in the VMHN and deep temporal isocortex; however, the number of PAPs and RTKs was directly proportional to the axon density. Interestingly, the PAP/RTK ratio was comparable between the three brain areas. At a time that we ignore the actual functional meaning of either type of process, their coexistence and association with the disrupted and intact myelin envelope, respectively, lead to infer that they assume opposite, yet, interrelated roles in myelin turnover.

A fundamental feature of glial cells is their synergic involvement in myelin genesis. Thus, ACs influence the onset of ontogenetical myelination by induction of the precursor cell to the myelin- synthesizing Ol (see Clemente et al., 2013). Furthermore, during adulthood, both cell types are united by gap junctions for which the existence of a functional glial framework has been supported (Black and Waxman, 1988; Orthmann-Murphy et al., 2008). In this context, previous and present observations demonstrating that both cell types interact directly with the myelin envelope opens the possibility that myelin remodeling results from a likewise, dynamically balanced interaction (Clemente et al., 2013; Zorec et al., 2017). In view of the absence of myelin phagocytosis or lysosome secretion by the normal Ol, it has been accepted that myelin renewal involves the molecular sorting and trafficking at sites of the Ol-myelin continuity (see Maier et al., 2008). In consonance with this, we found no altered myelin sheaths at sites of Ol-myelin juxtaposition and PAP-RTK frequencies, although different in magnitude, paralleled to each other. Given the signed association of PAPs with the disrupted myelin envelope and that of the Ol-RTK with the intact myelin sheath, we may infer that they represent putative substrata for a balanced myelin renewal and removal, respectively. Results of our ongoing experimental trials testing possible involvement of glial processes in response to axonal damage should enhance the likelihood of the latter postulation.

## Ethics statement

All experiemntal animals were males of 10 weeks of age that were raised in a pathogen-free colonies in accordance with animal care policies in our vivarium. An ad hoc ethics committee on animal experimentation approved the manipulation and sacrifice of animals, which was carried out according to the Animal Research Committee guidelines of the Instituto de Neurobiología.

## Author contributions

AV designed the light microscopic study, performed observations at the confocal microscope and wrote the manuscript's resulting section. VB retrieve the electron microscopic images and performed morphometry and 3D reconstructions. CL realized the inmuno- and histo-chemical techniques and processed images and videos from confocal microscopy. JL, designed the study, performed observations, illustrations, and wrote the final version of the manuscript.

### Conflict of interest statement

The authors declare that the research was conducted in the absence of any commercial or financial relationships that could be construed as a potential conflict of interest.
